# Synthesis, electrochemical properties, and antioxidant activity of sterically hindered catechols with 1,3,4-oxadiazole, 1,2,4-triazole, thiazole or pyridine fragments

**DOI:** 10.3762/bjoc.20.202

**Published:** 2024-09-19

**Authors:** Daria A Burmistrova, Andrey Galustyan, Nadezhda P Pomortseva, Kristina D Pashaeva, Maxim V Arsenyev, Oleg P Demidov, Mikhail A Kiskin, Andrey I Poddel’sky, Nadezhda T Berberova, Ivan V Smolyaninov

**Affiliations:** 1 Chemistry Department, Astrakhan State Technical University, Tatischev str. 16/1, 414056, Astrakhan, Russia; 2 G.A. Razuvaev Institute of Organometallic Chemistry, Russian Academy of Sciences, Tropinin str. 49, 603137, Nizhny Novgorod, Russia; 3 North-Caucasus Federal University, Pushkin str. 1, 355017, Stavropol, Russia; 4 Kurnakov Institute of General and Inorganic Chemistry, Russian Academy of Sciences, Leninskii prosp., 31, 119991, Moscow, Russia; 5 Institute of Inorganic Chemistry, University of Tübingen, Auf der Morgenstelle 18, 72076, Tübingen, Germany

**Keywords:** antioxidant activity, catechol thioethers, heterocycles, redox-transformations, thiones

## Abstract

A series of new RS−, RS−CH_2_− and R_2_N−CH_2_-functionalized сatechols with heterocyclic fragments such as 1,3,4-oxadiazole, 1,2,4-triazole, thiazole, or pyridine were synthesized by the reaction of 3,5-di-*tert*-butyl-*o*-benzoquinone or 3,5-di-*tert*-butyl-6-methoxymethylcatechol with different heterocyclic thiols. The S-functionalized catechols were prepared by the Michael reaction from 3,5-di-*tert*-butyl-*o*-benzoquinone and the corresponding thiols. The starting reagents such as substituted 1,3,4-oxadiazole-2-thiols and 4*H*-triazole-3-thiols are characterized by thiol–thione tautomerism, therefore their reactions with 3,5-di-*tert*-butyl-6-methoxymethylcatechol can proceed at the sulfur or nitrogen atom. In the case of mercapto-derivatives of thiazole or pyridine, this process leads to the formation of the corresponding thioethers with a methylene linker. At the same time, thiolated 1,3,4-oxadiazole or 1,2,4-triazole undergo alkylation at the nitrogen atom in the reaction with 3,5-di-*tert*-butyl-6-methoxymethylcatechol to form the corresponding thiones. The yield of reaction products ranges from 42 to 80%. The crystal structures of catechols with 3-nitropyridine or 1,3,4-oxadiazole-2(3*H*)-thione moieties were established by single-crystal X-ray analysis. The possibility of forming intra- and intermolecular hydrogen bonds has been established for these compounds. The electrochemical behavior of the studied compounds is influenced by several factors: the nature of the heterocycle and its substituents, the presence of a sulfur atom in the catechol ring, or a thione group in the heterocyclic core. The radical scavenging activity and antioxidant properties were determined using the reaction with synthetic radicals, the cupric reducing antioxidant capacity assay, the inhibition process of superoxide radical anion formation by xanthine oxidase, and the process of lipid peroxidation of rat liver (*Wistar*) homogenates in vitro.

## Introduction

Synthetic derivatives of polyphenols, in particular catechol (hydroquinone), represent a promising group of pharmacologically active substances [1–2]. Catechol-containing compounds demonstrate neuroprotective, antihypoxic effects, act as antiparkinsonian agents [3–5], exhibit antitumor and antibacterial activity [6–8], possess antioxidant properties for regulating free radical processes [9–11]. The functionalization of polyphenolic compounds by introducing various substituents or heteroatoms (nitrogen, sulfur, selenium, tellurium, etc.) as well as redox-active functional groups allows one to vary significantly the biological activity of such compounds.

Heterocyclic molecular blocks are widely used in medicinal chemistry [12]. Thiazole, oxadiazole, triazole, imidazole, and other heterocycles are classified as privileged medicinal scaffolds being components of many drugs [13–14]. Thiazole derivatives and their reduced forms exhibit antitumor (thiazofurin, ixabepilone), antibacterial (cefotaxime, ceftaroline, cefiderocol), antifungal (isavuconazole, fosravuconazole), antiviral (simeprevir, ritonavir, cobicistat), and other types of biological activities [15–19]. Besides, among the registered sulfur-containing drugs, 19% contain a thiazole ring, and 15% contain a thioether group [20]. Oxadiazole fragments are found in the structures of anticancer drugs (zibotentan), antituberculosis agents [13], as well as in drugs against arrhythmia (nesapidil) or muscular dystrophy (ataluren) [21]. The 1,3,4- and 1,2,4-oxadiazoles are the most promising from the point of view of bioactivity among the four isomers of oxadiazole. The presence of a 1,3,4-oxadiazole ring in the structure of compounds leads to an increased lipophilicity and, as a result, facilitates the transfer of the molecule through cell membranes to the target site [22]. 1,2,4-Triazole derivatives also play a key role in medicinal chemistry, especially in the development of new antivirals [23–24] and antibacterial agents [25].

A feature of mercapto-substituted heterocycles is the possibility of their existence in two tautomeric forms: thiol or thione [26–27]. The ability to tautomeric transformations affects their biological activity and allows the use of these molecular blocks to design compounds with different properties [28]. Anti-tuberculosis activity was identified for 5-substituted 2-mercapto-1,3,4-oxadiazoles [29], 5-substituted-2-[(3,5-dinitrobenzyl)-sulfanyl]-1,3,4-oxadiazoles and 1,3,4-thiadiazoles [30]. Some derivatives of 5-(4-*tert*-butylphenyl)-1,3,4-oxadiazole-2(3*H*)-thione showed antiproliferative activity against the HeLa cancer cell line [22]. 1,2,4-Triazole thioglycoside derivatives have antiviral activity against influenza strains H3N2 and H5N1 [31–32]. The 1,2,4-triazole-3-thione-imidazole hybrids displayed potential antibacterial activity against *E. coli*, *S. aureus* [33]. Heterocyclic thione derivatives are used as ligands in the synthesis of metal complexes (Ni(II), Pd(II), Pt(II), Cu(I), Ag(I) etc.) exhibiting antibacterial and antitumor activity [34].

In most cases, polyfunctional catechol thioethers were obtained by Michael reaction via the interaction of *o*-, *p*-benzoquinone and the corresponding thiol [35–38], in the nucleophilic substitution reaction in the aromatic ring of catechol [39–40] or under electrochemical conditions [41–43]. An anodic activation of catechols in the presence of a thiol leads to S-functionalized catechols with triazole, triazine, pyrimidine, imidazole, thiadiazole, or other fragments [44–48].

Previously, we obtained a series of sterically hindered catechols linked through a sulfide bridge with various polar or low-polar groups [36,49–51] and heterocyclic fragments [52] via the Michael addition reaction. Also, we have previously found that the reaction between 3,5-di-*tert*-butyl-6-methoxymethylcatechol and different functionalized thiols occurs via an acid-catalyzed mechanism and leads to corresponding thioethers with methylene linker [53–54]. Hybrid structures of this type are of particular interest from the point of view of studying their potential biological activity.

This work aimed to obtain new multifunctional catechols containing pharmacologically active heterocyclic fragments such as thiazole, 1,3,4-oxadiazole, 1,2,4-triazole, and pyridine. We decided to use a non-hydrolyzable thioether group as a bridging moiety. A feature of heterocyclic thiols used as starting reagents is the possibility of thiol–thione tautomerism which leads to the appearance of two nucleophilic centers: a sulfur or nitrogen atom. Therefore, in the case of their interaction with 3,5-di-*tert*-butyl-6-methoxymethylcatechol, the possibility of alkylation of the nitrogen atom in the heterocycle cannot be excluded. This alternative pathway is similar to the previously described interaction of the aforementioned catechol with 3,5-dimethylpyrazole or benzimidazole [55]. A study of the structure, electrochemical properties, anti/prooxidant, and antiradical activity was carried out for the catechols synthesized in this work.

## Results and Discussion

### Synthesis

**Scheme 1:**
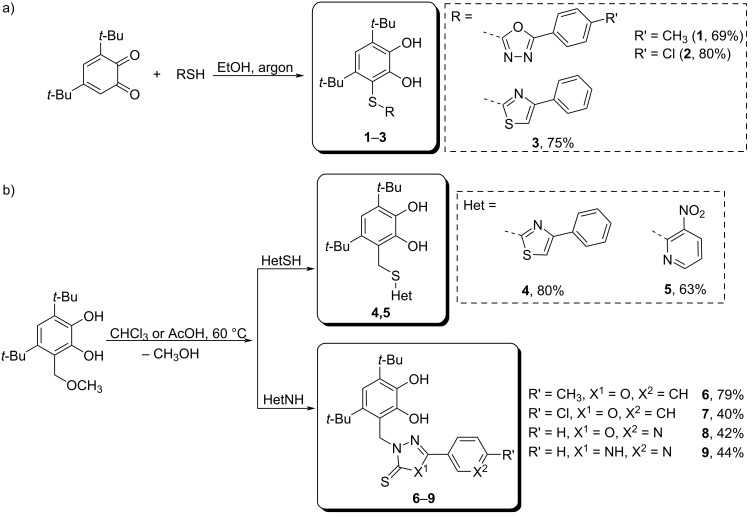
Synthesis of catechol-containing compounds **1**–**9**.

The interaction of 3,5-di-*tert*-butyl-*o*-benzoquinone with the corresponding thiols in ethanol at room temperature under argon leads to the formation of catechol thioethers **1**–**3** (69–80%) (Scheme 1a). The reaction between 3,5-di-*tert*-butyl-6-methoxymethylcatechol and 2-mercapto-4-phenylthiazole or 3-nitropyridine-2-thiol occurs via an acid-catalyzed mechanism and leads to corresponding thioethers **4**, **5** with methylene linker in a good yield (80% and 63%, correspondingly) (Scheme 1b). At the same time, the interaction of 2-mercapto-substituted derivatives of 1,3,4-oxadiazole or 1,2,4-triazole with the starting catechol proceeds at the nitrogen atom of the five-membered heterocycle due to the possibility of the thione–thiol tautomerism in the starting thiol [26–27]. Consequently, compounds **6**–**9** are products of alkylation of the nitrogen atom of the heterocycle. Thiones **6**–**9** were obtained in 40–79% yield (Scheme 1,b).

The structures of synthesized compounds were confirmed by the spectral methods IR-, ^1^H NMR, ^13^C{^1^H} NMR spectroscopy (Figures S1–S18 in Supporting Information File 1), HRMS, and elemental analysis. The molecular structures of compounds **5**, **6** and **8** in crystal state were determined by single-crystal X-ray analysis.

### X-ray data

**Figure 1:**
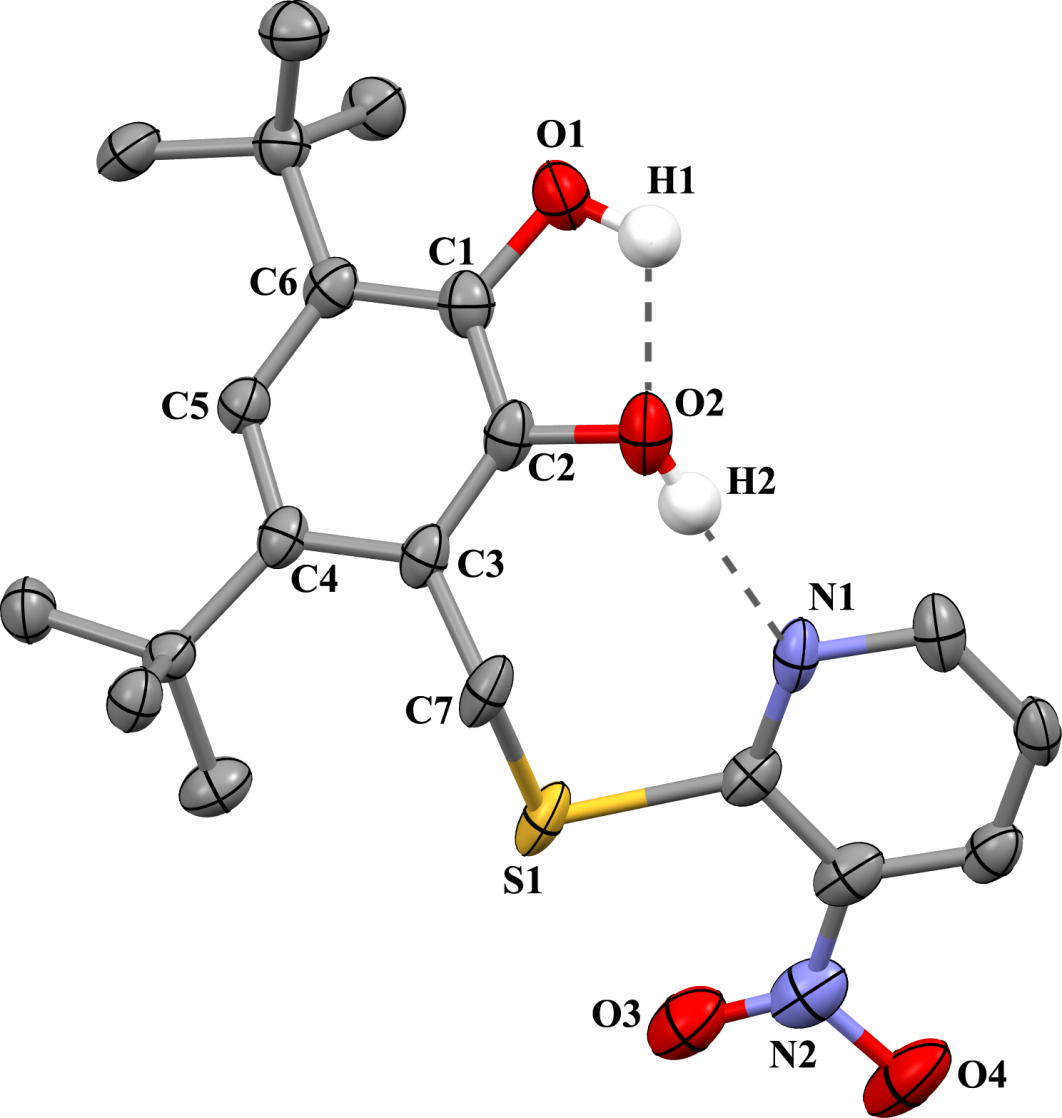
The X-ray structure of catechol **5** (the thermal ellipsoids of 50% probability). The hydrogen atoms except those of hydroxy groups are omitted.

**Figure 2:**
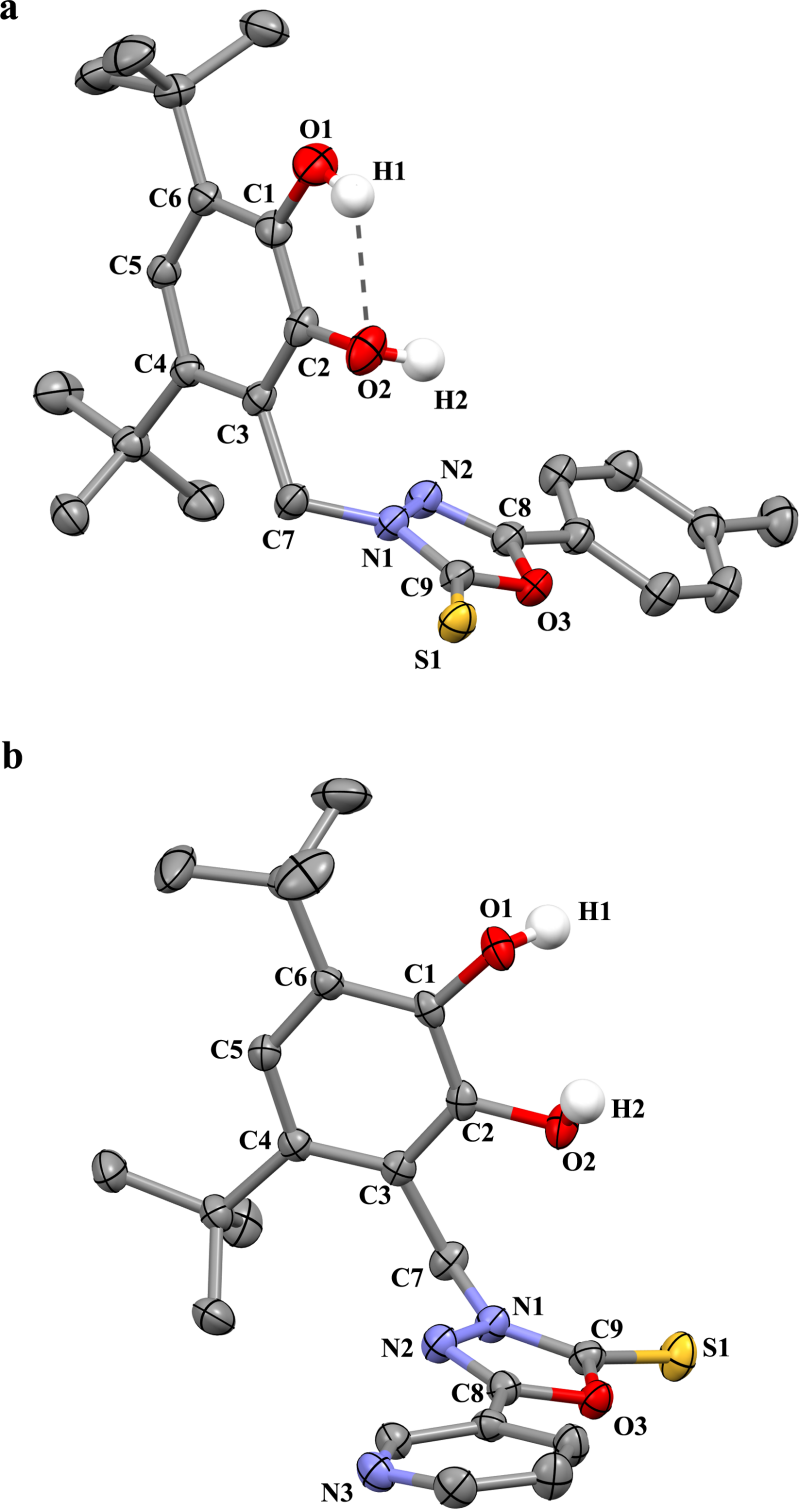
The X-ray structures of catechols **6** (a) and **8** (b) (the thermal ellipsoids of 50% probability). The hydrogen atoms except those of hydroxy groups are omitted.

The X-ray suitable crystals **5**, **6**·0.5CH_3_CN, and **8** were grown by slow recrystallization of the compounds from acetonitrile solutions. The unit cells of complexes **5** and **8** do not contain any solvent molecules, while the unit cell of crystals **6**·0.5CH_3_CN contains one acetonitrile molecule per two catechol molecules. The nitro-substituted pyridine group in **5** is disordered in two positions. The X-ray structure of catechol **5** is shown in Figure 1, and the X-ray structures of catechols **6**, and **8** are shown in Figure 2.

The geometrical characteristics of catechol moieties of these compounds, in general, have the standard parameters: the bonds C1–O1, C2–O2 are 1.369(11) and 1.377(9) Å in **5**, 1.371(2) and 1.387(2) Å in **6**, 1.389(3) and 1.377(3) Å in **8**; the average lengths of the С–С bonds of six-membered carbon cycle С(1–6) (1.396 ± 0.013 Å in **5**, 1.397 ± 0.009 Å in **6**, 1.396 ± 0.012 Å in **8**) are typical for catechols [50,55–56].

The presence of complex polyfunctional substituents affects their spatial location regarding a catechol fragment. In most cases, the catechol molecules in the crystal are located in such a way that catechol hydroxy fragments look at each other and, in addition to intra-molecular hydrogen bond O–H···O between the two hydroxy groups, the intermolecular hydrogen interactions are also being realized between the catechol fragments of neighboring molecules [57–61]. The intra- and intermolecular interactions in crystals **5**, **6**·0.5CH_3_CN, and **8** lead to different complex structural motifs in crystals of these compounds.

Two intramolecular hydrogen bonds (Figure 1) are observed in the crystal structure of **5**: between OH groups (O1–H1···O2: the H1···O2 distance is 1.98(1) Å, the O1···O2 distance is 2.581(3) Å, angle O1–H1–O2 is 117°) and between hydroxy group O2–H2 and nitrogen atom N1 of the pyridine cycle (O2–H2···N1A/B: the H2···N1 distance is 1.75(1)/2.08(1) Å, the O2···N1 distance is 2.560(3)/2.893(3) Å, angle O2–H2–N1 is 160°/164°, here and further, two data sets are given, taking into account the pyridine cycle disorder).

**Figure 3:**
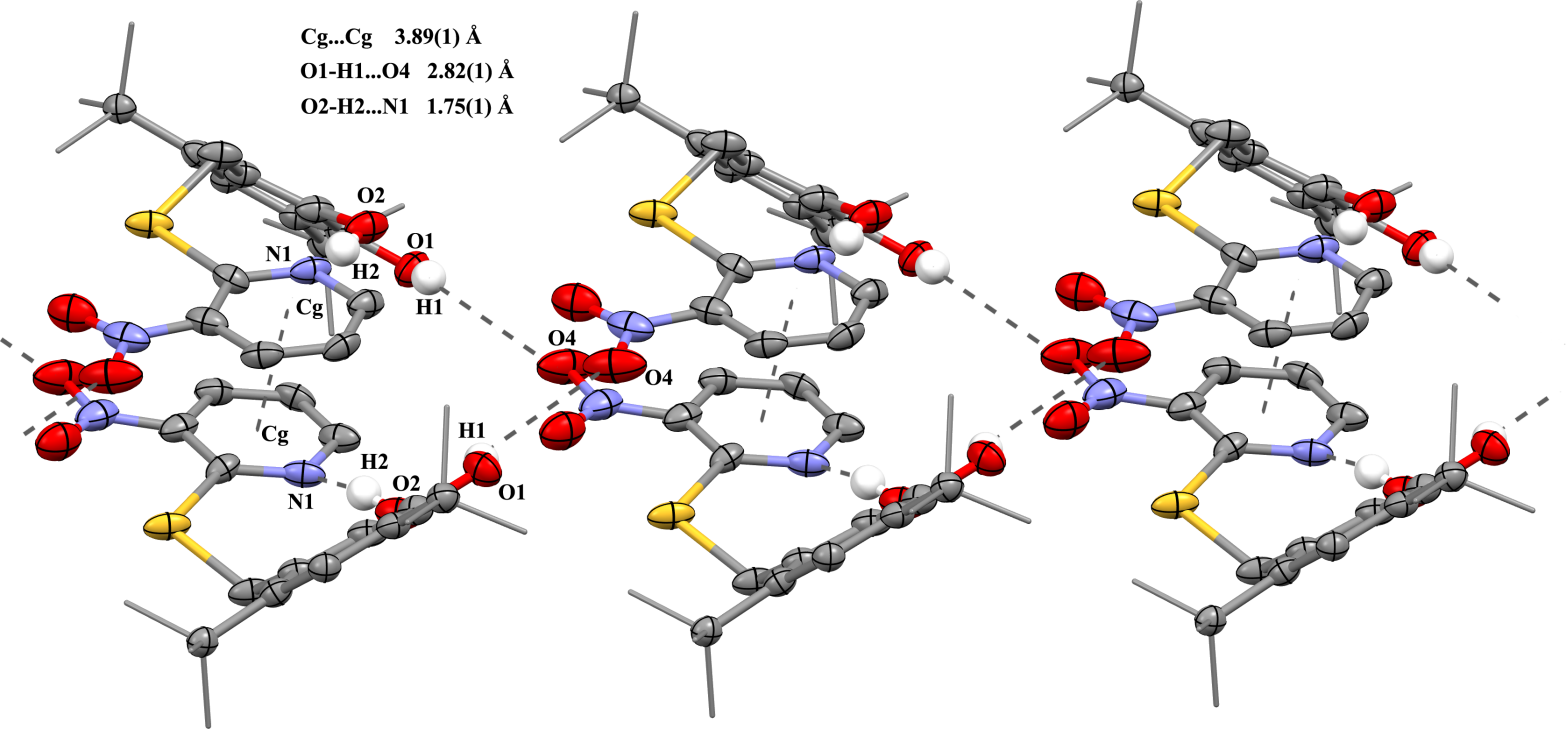
Fragment of the pack of catechol **5** in crystal (the H-bonds and π–π interactions are shown as dotted lines).

In the crystal of **5**, two molecules form pairs due to the π–π interactions between pyridine cycles (the Cg···Cg distance between centroids is 3.855(4) and 3.891(4) Å; the angle between the planes of the cycles corresponds to 5°). These pairs (Figure 3) are bound in a supramolecular chain along the 0*c* axis owing to two H-bonds between the OH group of catechol and the oxygen atom of nitro-group (O1–H1···O4B (1−*x*, 1−*y*, −1+*z*): the H1···O4 distance is 2.46(1) Å, the O1···O4 distance is 2.886(3) Å, the angle O1–H1–O4 106°; O1–H1···O4A: the H1···O4 distance is 2.82(1) Å, the O1···O4 distance is 3.146(3) Å, the angle O1–H1–O4 101°, Figure 3). Additionally, the packing of these chains in the crystal is caused by the intermolecular C–H···O and N–O···π contact.

**Figure 4:**
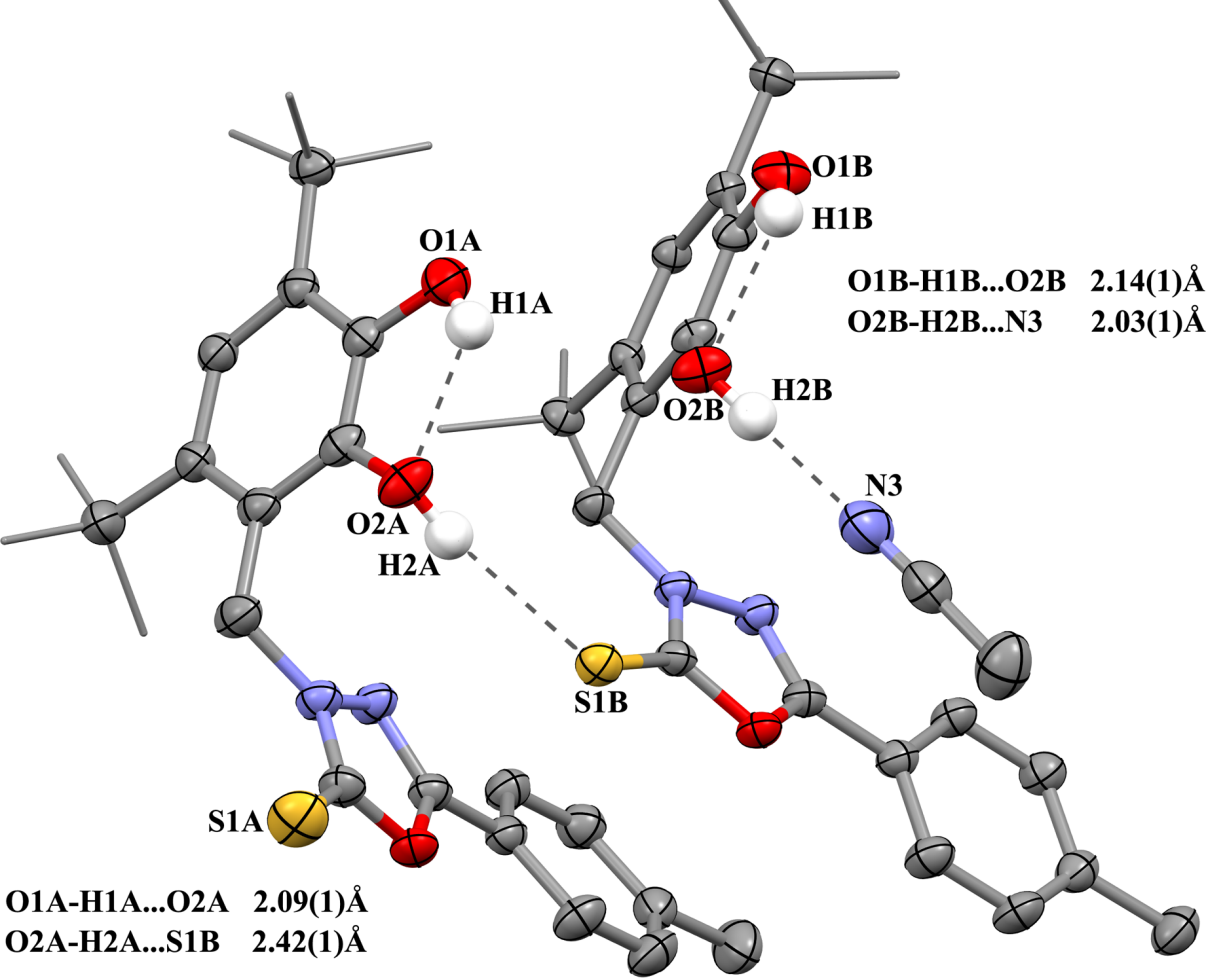
The interactions in pair of independent molecules A and B of **6** in crystal **6**·0.5CH_3_CN (the H-bonds are shown as dotted lines).

Catechol **6** in crystal also forms pairs formed by two crystallographically independent molecules А and В (Figure 4). Intermolecular hydrogen interaction O1–H1···O2 takes place in both molecules of this pair (the distance H1A···O2A is 2.09(1) Å, the angle O1A–H1A–O2A is 122°; the distance H1B···O2B is 2.14(1) Å, the angle O1B–H1B–O2B is 121.6°). In addition, the intermolecular H-bonding is observed between group O2–H2 of molecule A and the thione group C=S of molecule B (the distance H2A···S1B is 2.42(1) Å, the angle O2A–H2A–S1B is 161°).

At the same time, for molecule B, there is a hydrogen bond between the O2B–H2B group with the nitrogen atom N3 of the nitrile group of the solvent acetonitrile molecule presenting in the cell: the corresponding distance H2B···N3 is 2.03(1) Å, the angle O2B–H2B–N3 is 169°. As a result, molecules A and B in this pair are located according to the principle of "chairs inserted into each other."

**Figure 5:**
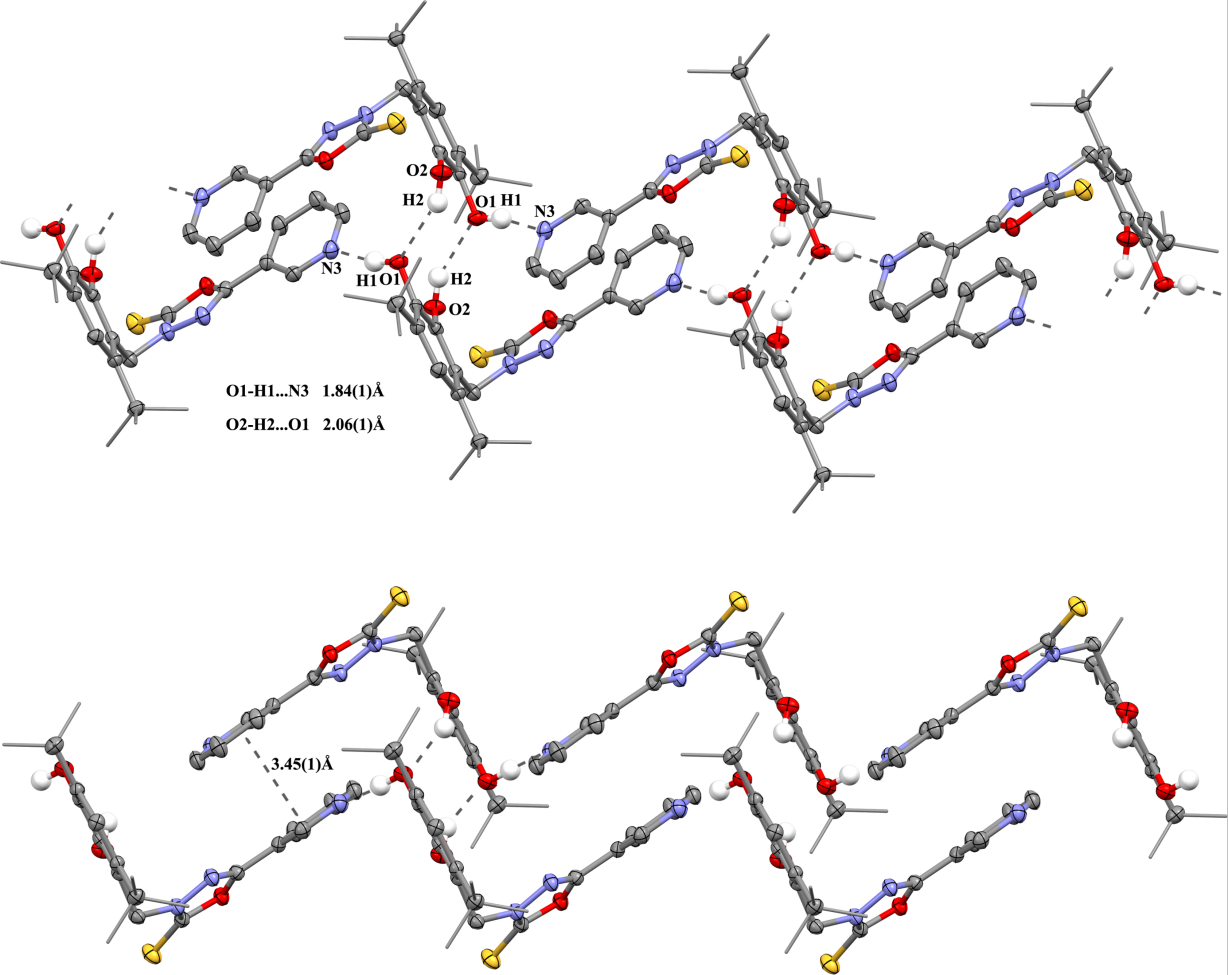
Fragment of the pack of catechol **8** in crystal (the H-bonds and π–π interactions are shown as dotted lines).

The intermolecular H-bonding O2–H2···O1 between catechol moieties of two neighbouring molecules leading to pairs was also observed in the crystal of catechol **8** (Figure 5): The H2···O1 distance is 2.06 (1) Å, the O2···O1 distance is 2.87(1) Å, the angle O2–H2–O1 is 151.4°. However, the additional intermolecular contacts O1–H1···N3 (the H1···N3 distance is 1.84(1) Å, the O1···N3 distance is 2.73(1) Å, the angle O1–H1–N3 is 168.3°) with the pyridine group of the catechol moiety of the neighbouring pair lead to the formation of chains. As a result of such interaction, the hydroxy group O1–H1 is turned away from the neighbouring group O2–H2, and there is no intramolecular hydrogen bond between hydroxy groups in molecule **8** (Figure 2(b)). A π-stalking was found between the pyridine groups in molecules belonging to the neighbouring pairs with the corresponding distance of 3.45(1) Å.

### Electrochemical properties

The study of the electrochemical properties of functionalized catechols allows one to suggest the mechanism of their electrooxidation, establish electron transfer centers, and predict antioxidant activity based on electrochemical data. To determine electron-transfer centers, the oxidation potentials of the starting mercapto-substituted heterocycles were studied. Based on cyclic voltammetry data, the starting compounds can be arranged in increasing order of the anodic peak potentials: 5-(3-pyridyl)-1,2,4-triazole-3-thiol (0.87 V) < 2-mercapto-4-phenylthiazole (0.94 V) < 3-nitropyridine-2-thiol (1.18 V) < 1,3,4-oxadiazole derivatives (1.37–1.39 V). The redox properties of catechols **1**–**9** were studied by cyclic voltammetry (CV) (Table 1).

**Table 1:** The peak potentials of **1**–**9** obtained by the CV (GC-electrode, CH_3_CN, *ν* = 0.2 V∙s^−1^, 0.1 М *n*-Bu_4_NClO_4_, *c* = 1–3 mmol·L^−1^, Ag/AgCl/KCl (sat.)).

Compound	**1**	**2**	**3**	**4**	**5**	**6**	**7**	**8**	**9** ^a^

*E*^pa1^, V	1.25	1.22	1.02	0.94	1.00	1.17	1.19	1.17	0.85
*E*^pa2^, V	1.56	1.55	1.81	1.56	1.84	1.64	1.68	1.90	–

^a^Compound was dissolved in MeCN/DMSO = 3:1 (v:v).

**Scheme 2:**
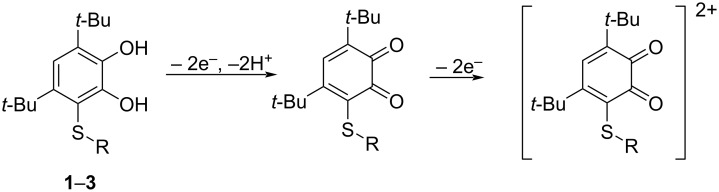
Electrochemical transformations of compounds **1**–**3**.

In the anodic area, the electrochemical profile for catechol thioethers **1**–**5** represents two successive irreversible oxidative stages. The first two-electron peak is observed in the range of 0.94–1.25 V. It refers to the oxidation of the catechol moiety to the corresponding *o*-benzoquinone, as previously shown for related compounds [36]. The second redox transition at 1.55–1.84 V characterizes the oxidation of the sulfide fragment (Scheme 2).

To confirm the participation of the catechol group in the first redox transition, the microelectrolysis of **3** was carried out at a controlled potential of 1.35 V in MeCN (2 h, 0.8 F/mol). After electrolysis, a decrease in the current intensity of the first oxidation peak is observed on the CVs of this compound (conversion reaches 51%). Besides, the current value of the second peak remains unchanged. In the cathodic region, a one-electron quasi-reversible peak is displayed (*E*_pc_ = −0.58 V). This process corresponds to the reduction of *o*-benzoquinone to *o*-benzosemiquinone. During electrolysis, the color of the solution changes. This is confirmed by the appearance of an absorption maximum in the UV–visible spectrum at λ = 490 nm, which is characteristic of thiolated *o*-benzoquinones. This absorption band corresponds to intramolecular charge transfer between the thioether group and the *o*-benzoquinone fragment [36,62–65].

**Figure 6:**
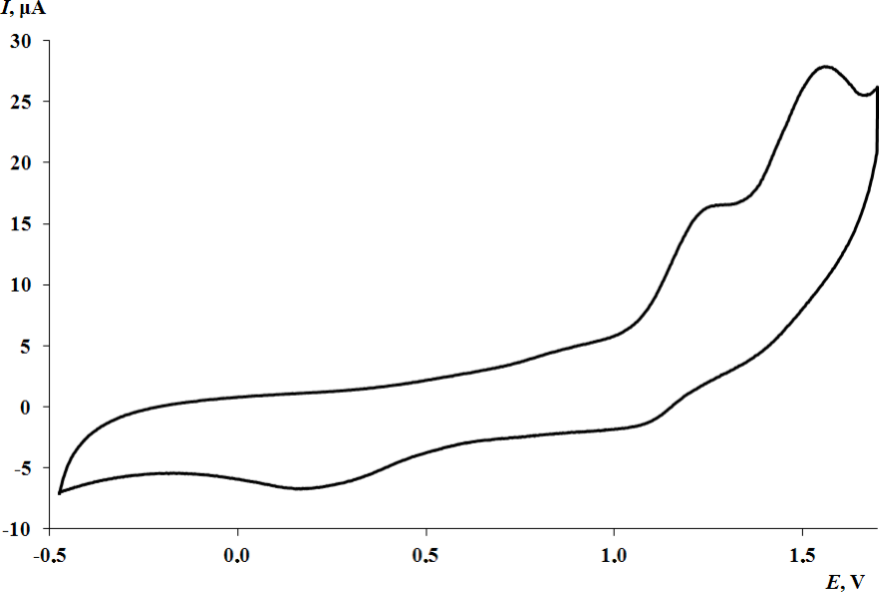
The CV curve of **2** at the potential range from −0.50 to 1.60 V (CH_3_CN, GC electrode, Ag/AgCl/KCl(sat.), 0.15 M *n*-Bu_4_NClO_4_, *c* = 1 mmol·L^−1^).

The potentials of the first anodic peak of thioethers **1** and **2** with an oxadiazole core are fixed at close values. (Supporting Information File 1, Figure S19 for **1**, Figure 6 for **2**).

**Figure 7:**
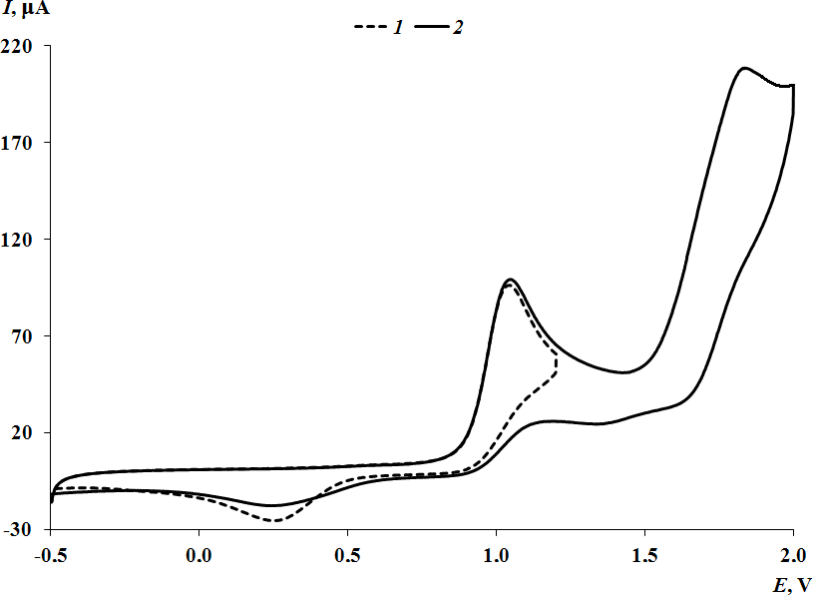
The CV curves of **3** at the potential ranges from –0.5 to 1.2 V (curve 1); from –0.5 to 2.0 V (curve 2) (CH_3_CN, GC electrode, Ag/AgCl/KCl(sat.), 0.15 M *n*-Bu_4_NClO_4_, *c* = 3 mmol·L^−1^).

The replacement of the oxadiazole cycle with a thiazole one in catechol thioether **3** leads to a shift in the oxidation peak of the catechol fragment to the cathode region (Figure 7). This is due to the less acceptor nature of the thiazole ring compared to the more electron-deficient oxadiazole cycle, which contains three heteroatoms. In addition, according to NMR spectral data, one proton of the hydroxy group of catechol **3** is shifted to the thiazole nitrogen atom, which also affects the decrease in the oxidation potential. In the case of **3**, the second anodic peak corresponding to thioether oxidation is multielectronic and shifted towards more positive values.

More intensive current can be explained by the proximity of the thioether linker and the thiazole moiety oxidation potentials. The appearance of a methylene bridge between the aromatic ring and the sulfur atom in catechol **4** leads to a shift of the catechol oxidation peak to the cathodic area (Supporting Information File 1, Figure S20) as compared to catechol **3**. This effect is due to a decrease in the acceptor effect of the thioether group. In turn, the second two-electron stage for **4** is recorded at earlier potential values. On the contrary, this behavior is caused by the presence of CH_2_-linker which divides electrogenerated quinone form and heterocycle moiety. The formation of intramolecular hydrogen bonds between the OH group and the pyridine ring facilitates the electrooxidation process (1.,0 V) (Supporting Information File 1, Figure S21) for catechol thioether **5**. The second oxidation peak is shifted to the anodic region due to the influence of the electron-withdrawing nitro group.

**Figure 8:**
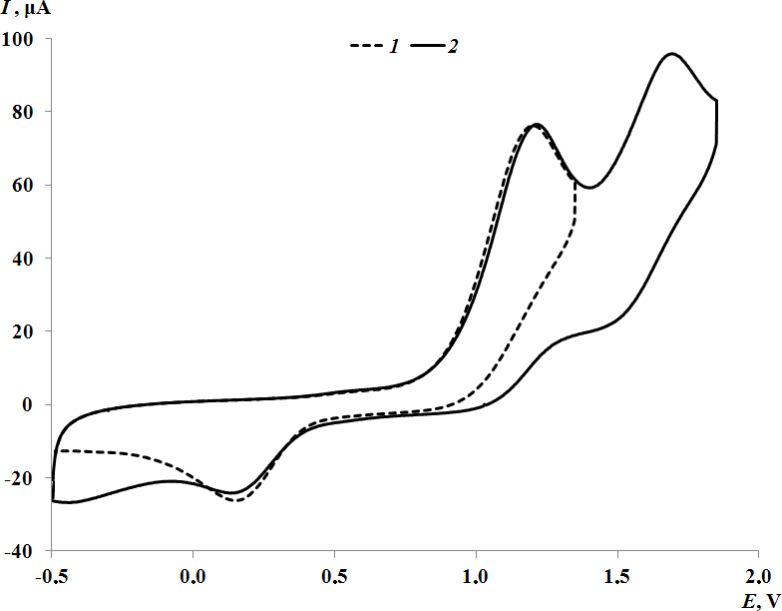
The CV curves of **7** at the potential ranges from –0.5 to 1.3 V (curve 1); from –0.5 to 1.8 V (curve 2) (CH_3_CN, GC electrode, Ag/AgCl/KCl(sat.), 0.15 M *n*-Bu_4_NClO_4_, *c* = 3 mmol·L^−1^).

Some differences are observed in the electrochemical behavior of thiones **6**–**9** compared to thioethers **1**–**5**. In the case of compounds **6**–**8** with a 1,3,4-oxadiazole-2-thione fragment, the oxidation potential of the first peak is shifted to the cathodic region compared to catechols **1** and **2** which contain a similar heterocycle (Figure 8 for **7**, Supporting Information File 1, Figures S22 and S23 for **6** and **8**, respectively). This effect is due to the presence of a CH_2_ linker which separates the catechol and heterocyclic fragments, thereby reducing the acceptor effect of the heterocycle on the catechol ring. The change of substituents in the 1,3,4-oxadiazole-2-thione ring has virtually no effect on the potential of the first redox transition. For compounds **6** and **7**, the second anodic peak is single-electron and presumably corresponds to the oxidation of the thione group. This signal is shifted to the anodic region by 0.09–0.13 V compared to the *E*^pa2^ values corresponding to the oxidation of the thioether group in catechols **1** and **2** with a similar heterocyclic fragment (Figure 6).

**Scheme 3:**
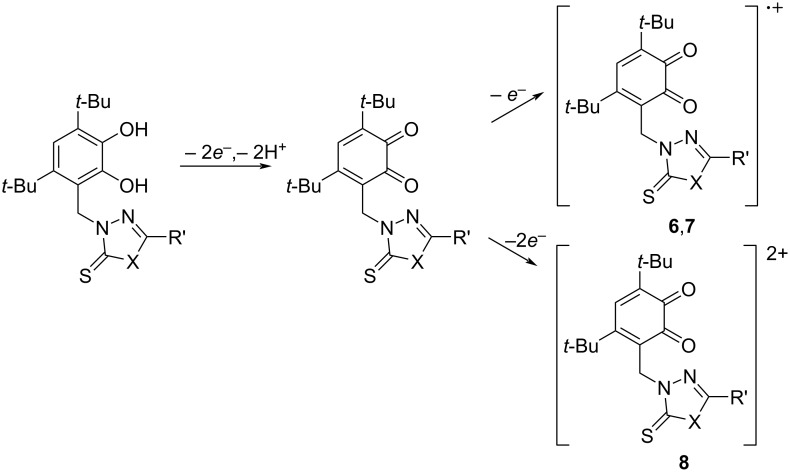
Proposed mechanism of an electrooxidation of compounds **6**–**8**.

The presence of an acceptor 3-pyridyl ring in the 1,3,4-oxadiazole-2-thione core for compound **8**, leads to a shift of *E*^pa2^ by 0.22–0.26 V to the anodic region compared to compounds **6** and **7**. In addition, the current intensity of the second peak corresponding to the transfer of two electrons increases (Figure S23 in Supporting Information File 1). The electrochemical behavior of catechol **9** with a 1,2,4-triazole-5-thione ring was studied in a MeCN–DMSO mixture to increase its solubility. The first redox transition is detected at early potential values (0.85 V) which coincides with the value obtained for the corresponding starting thiol. However, there is a peak at *E* = −0.11 V on the reverse CV branch of **9**, characteristic of compounds with a catechol fragment (Figure S24 in Supporting Information File 1). The second anodic peak for **9** is not observed due to the narrow electrochemical window of the solvent in the positive CV region. The formation of *o*-benzoquinones at the first stage of oxidation was confirmed during a controlled potential microelectrolysis of compound **6** at 1.30 V (2 h, 0.8 F/mol). A similar electrochemical picture is observed for electrolysis products of catechol **6** as in the case of catechol **3**. The recorded absorption spectra after the electrolysis display an intense maximum at λ = 412 nm which is characteristic for sterically hindered *o*-benzoquinones. The proposed scheme for the electrooxidation of catechols **6**–**9** is presented in Scheme 3.

Based on the current intensities of the second anodic peaks for compounds **6**–**8** with a 1,3,4-oxadiazole-2-thione ring (Figure 8 for **7**, Supporting Information File 1, Figures S22 and S23 for **6** and **8**, respectively), it can be assumed that the second redox transition can occur in one (for **6** and **7**) and a two-electron (for **8**) manner.

Thus, the electrochemical behavior of the studied catechols with heterocyclic fragments is influenced by several factors: the nature of the heterocycle and its substituents, the presence/absence of a sulfur atom in the catechol ring or thione sulfur atom in the heterocyclic core. Based on electrochemical data, it can be assumed that the most pronounced antioxidant activity can be exhibited by compounds with minimal first oxidation potentials: catechol **4** containing a 4-phenylthiazole ring and catechol thione **9** with a 1,2,4-triazole ring.

### Radical scavenging, anti/prooxidant activity of catechols with heterocyclic fragments

The radical scavenging properties and antioxidant activity of catechols **1**–**9** were investigated in the reaction with a diphenylpicrylhydrazyl (DPPH) radical, ABTS^·+^ radical cation, CUPRAC test, and inhibition process of superoxide radical anion formation by xanthine oxidase (NBT assay). The presence of a catechol fragment and thioether or thione groups determines the ability to neutralize different radical species. A comparative evaluation of the antioxidant activity of catechols **1**–**9** and Trolox was performed in a reaction with DPPH in dichloromethane at 298 K (Table 2).

**Table 2:** Radical scavenging activity characteristics of catechols **1–9** and Trolox in DPPH (CH_2_Cl_2_, 298 K), ABTS^•+^ and CUPRAC assays expressed as IC_50_ and TEAC.

Compound	DPPH		ABTS^∙+^	CUPRAC
	IC_50_ (DPPH), µM	TEC_50_, min	IC_50_ (ABTS^∙+^), µM	CUPRAC_TEAC_

**1**	>100	–	45.6 ± 1.8	0.12^a^
**2**	34.6 ± 1.3	40	38.4 ± 1.5	0.12
**3**	13.0 ± 1.0	13	17.6 ± 1.3	1.04
**4**	14.3 ± 1.5	15	17.9 ± 1.7	1.15
**5**	14.8 ± 0.8	35	25.3 ± 0.6	0.96
**6**	24.0 ± 1.1	15	10.5 ± 0.3	0.99
**7**	19.6 ± 2.0	20	11.6 ± 0.5	1.13
**8**	16.6 ± 0.8	25	20.6 ± 2.3	0.96
**9**	18.6 ± 0.7	17.0	13.9 ± 1.0	1.92^a^
Trolox	12.0 ± 0.5	10.3	16.0±1.0	1.00
CatH_2_-CH_2_-S-thiazolyl^b^	21.5 ± 1.5	<1.0	21.6 ± 1.8	0.83 ± 0.06
CatH_2_-CH_2_-S-pyridinyl^b^	11.5 ± 0.4	23.0	17.6 ± 2.0	1.25 ± 0.11

^a^Data were observed in DMSO. ^b^Data were obtained from [54].

Based on the IC_50_ value, compound **3** with a 4-phenylthiazole ring linked through a sulfur atom to the catechol ring exhibits the highest activity (13.0 ± 1.0 µM) in the reaction with the DPPH radical. The number of converted DPPH molecules per one molecule of **3** is equal to two. Comparable IC_50_ values were obtained for catechol thioethers **4** and **5** with methylene bridge.

As in the case of compound **3**, catechol **4** contains a 4-phenylthiazole moiety but is a less active radical scavenger, probably due to the presence of a CH_2_−linker between the catechol and heterocyclic moieties. At the same time, the appearance of a phenyl substituent in the thiazole ring of **4** reduces the IC_50_ value (14.3 ± 1.5 µM) compared to the previously studied 3-((thiazol-2-ylthio)methyl)-4,6-di-*tert*-butylcatechol (CatH_2_-CH_2_-S-thiazolyl) (21.5 ± 1.5 µM) [54]. However, for CatH_2_-CH_2_-S-thiazolyl, the equilibrium state is reached in less than one minute which increases significantly the antiradical activity of AE compared to catechol **4**. On the contrary, the presence of a nitro group in the pyridine ring in catechol **5** reduces its antiradical activity compared to the unsubstituted 3-((pyridin-2-ylthio)methyl)-4,6-di-*tert*-butylcatechol (CatH_2_-CH_2_-S-pyridinyl). In the series of catechols **6–8** with a thione group in the 1,3,4-oxadiazole ring, the IC_50_ values decrease gradually from 24.0 to 16.6 µM, while an inverse relationship is observed for TEC_50_. Replacement of the methyl group with a chlorine atom in the phenyl ring of compound **7** leads to a decrease in the IC_50_ value compared to catechol **6**. The appearance of a pyridyl substituent in the oxadiazole core has an even more noticeable effect on reducing the IC_50_. Catechol thioether **2** containing a 1,3,4-oxadiazole ring showed the least neutralizing activity. Compound **1** was inactive in this test (IC_50_ >> 100 µM). TEC_50_ varies in the range of 13–40 min for all studied compounds. Based on the results of the calculation of the AE indicator, which takes into account the parameters EC_50_ and TEC_50_ [66], the studied compounds can be arranged in ascending order of neutralizing activity: **2** (0.7) < **5** (1.9) < **8** (2.4) < **7** (2.5) < **6** (2.7) < **9** (3.1) < **4** (4.7) < Trolox (5.1) < **3** (5.9). Thus, in each series of thioethers (**1**–**5**) or thiones (**6**–**9**), the leading compound can be identified: catechol **9** with 1,2,4-triazole-5-thione core and **3** with 4-phenylthiazole cycle, which is the only one of the studied compounds exceeding Trolox activity in this assay. Consequently, the presence of 1,2,4-triazole-5-thione or 4-phenylthiazole fragment favors the pronounced antiradical activity.

The use of ABTS radical cation to assess the antioxidant capacity of compounds is one of the widely used methods which is based on the transfer of an electron from the studied molecules to the acceptor [67]. The obtained IC_50_ values for synthesized catechols vary over a wide range from 10.5 to 45.6 μM. As in the case of the reaction with the DPPH radical, thioethers **1** and **2** possess a maximal IC_50_ value which indicates their low radical scavenging activity. Compounds **3** and **4** containing a 4-phenylthiazole moiety have similar IC_50_ values. This points out that the methylene bridge separating the catechol ring and the sulfur atom does not influence their neutralizing activity. The presence of a phenyl substituent in the heterocyclic fragment of compound **4** has a positive effect on the antiradical activity in this test in comparison with CatH_2_-CH_2_-S-thiazolyl. The electron-withdrawing NO_2_ group in the pyridine ring of catechol **5** suppresses its antiradical properties in comparison with CatH_2_-CH_2_-S-pyridinyl which is similar to the patterns observed in the DPPH test. In general, compounds with thione moieties were more active ABTS^·+^ scavengers than thioethers **1**–**5** except catechol **8**. The minimum IC_50_ values in this test were obtained for compounds **6** and **7**. Therefore, catechol thiones **6**, **7**, and **9** were more effective ABTS^·+^ scavengers than Trolox.

The CUPRAC-assay is used to determine the electronic capacity of an antioxidant relative to Trolox under the action of a soft oxidant a bis-(neo-cuproine) copper(II) complex. The CUPRAC_TEAC_ parameter varies in a wide range of values from 0.12 to 1.92 (Table 2). Compounds **1** and **2** containing a 1,3,4-oxadiazole ring and a sulfur atom directly bonded to the catechol ring are practically inactive in this reaction which correlates with the data of the DPPH-test. Compounds **3**, **5**, **6**, and **8** exhibit antioxidant activity comparable to Trolox, while catechols **4** and **7** are 13–15% higher than the activity of this standard. The introduction of an electron-withdrawing nitro-group into the pyridine ring leads to a 29% decrease in the CUPRAC_TEAC_ value for compound **5** compared to CatH_2_-CH_2_-S-pyridinyl. The presence of a phenyl substituent in the thiazole ring has a positive effect on the antioxidant activity of compound **4** and leads to its increase by 32% compared to the previously studied CatH_2_-CH_2_-S-thiazolyl. The highest CUPRAC_TEAC_ value (1.92) was determined for **9** with 3-(pyridin-3-yl)-1,2,4-triazole-5-thione fragment. Thus, in this test, thioether **4** and thiones **7** and **9** have the greatest antioxidant capacity.

The neutralizing activity of compounds **1**, **3**, **4**, and **8** concerning the superoxide anion radical was studied in the NBT-test. Based on the IC_50_ values, compound **8** with the 5-(pyridin-3-yl)-1,3,4-oxadiazole-2-thione fragment showed a pronounced inhibitory effect (31.13 ± 0.84 μM) compared to Trolox (92.55 ± 0.24 μM). Among compounds **3** and **4** containing a thiazole ring, the more effective O_2_^·–^ scavenger is catechol **3** (40.39 ± 0.89 μM), in which the sulfur atom is directly bonded to the aromatic ring. The IC_50_ value is 63.48 ± 1.08 μM for compound **4**. The appearance of a phenyl substituent in the thiazole ring affects negatively the antiradical ability of compound **4** compared to its unsubstituted analogue CatH_2_-CH_2_-S-thiazolyl (34.0 ± 0.10 μM). Compound **1** was the least effective in this test (81.10 ± 1.86 μM) as in the case of the methods described above.

**Figure 9:**
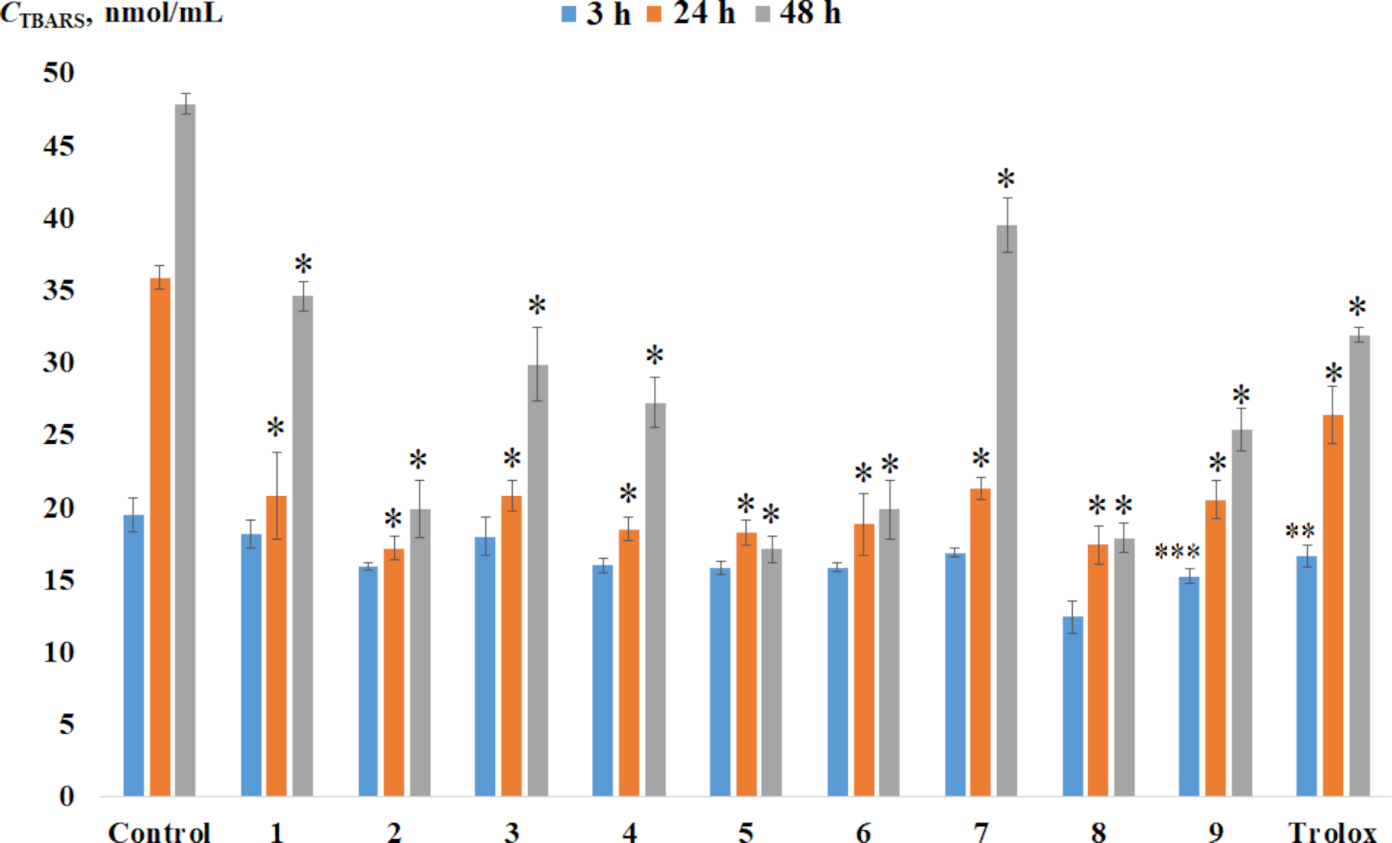
The level of TBARS in rat liver homogenates in vitro, in the presence of compounds **1**–**9**, Trolox, and without additives (control) during the incubation for 3, 24, and 48 hours (concentration of compounds in the reaction medium is 0.1 mM). The results are expressed as mean ± standard deviation (* p < 0.001; ** p < 0.01; ***p < 0.05).

We have previously shown that catechol thioethers can act as anti- or prooxidants on lipid peroxidation processes in vitro depending on a functional group or a heterocycle substitute at the sulfur atom [49–50,54]. Non-enzymatic process of the rat (Wistar) liver homogenate lipid peroxidation (LP) in the presence of additives of target compounds **1**–**9** was studied in vitro*.* The concentration of the carbonyl compounds forming in the reaction of the lipid peroxidation was determined by the accumulation of a colored complex with thiobarbituric acid (TBARS) (Figure 9).

A decrease in TBARS concentration was observed in the presence of all tested compounds which indicates their antioxidant effect during incubation time. At the initial stage (3 h), catechol thione **8** containing a pyridine substituent in the 1,3,4-oxadiazole-2-thione cycle has the greatest inhibitory effect (36%) on the LP process compared to the control sample. For compounds **2**, **4**–**7**, and **9**, a less pronounced antioxidant effect (13–19%) is observed comparable to the inhibitory activity of Trolox. Initially (3 h), catechols **1** and **3** exhibit a weak inhibitory effect (7–8%). An increase in the incubation time to 24 hours leads to a rise in antioxidant activity for all compounds. A decrease in TBARS content by 41–52% was noted compared to the control experiment. All the studied compounds exhibit more effective inhibitory activity than Trolox. The inhibitory effect for compounds **2**, **5**, **6**, **8**, and **9** increases to 47–64% after 48 hours. This indicates the ability of these catechols to have a prolonged effect on the LP process. In the presence of Trolox, amounts of TBARS decrease by 33% over 48 hours of incubation.

The *C*_TBARS_ value varies in a narrow range of values and is practically independent of experimental time for **5** and **6**. This behavior indicates the occurrence of an induction period during lipid peroxidation when the level of TBARS remains constant. In the case of **1** and **7**, a decrease in the inhibitory effect to 17–28% is observed with an increase in incubation time from 24 to 48 hours. Catechol thioether **5** with a 3-nitro-substituted 2-pyridine moiety and **8** with a 3-pyridine substituent in the 1,3,4-oxadiazole-2-thione ring demonstrate the maximum antioxidant activity (63–64%) over 48 hours of the experiment. A comparative assessment of the effect of thioethers **1** and **2** in the LP process points out that the replacement of the methyl group in the phenyl ring with a chlorine atom leads to increased antioxidant activity of **2**. In the presence of catechol thioether **3**, the TBARS content is slightly lower compared to **4**. A phenyl substitute in the thiazole ring in **4** affects negatively its antioxidant activity in the LP process compared to CatH_2_-CH_2_-S-thiazolyl reducing the inhibitory effect from 81% to 43% (48 h). Consequently, catechol thioethers **2** and **5** as well as catechol thione **8** are the most effective antioxidants in the lipid peroxidation reaction.

## Conclusion

Thus, we have synthesized a series of new sterically hindered catechols with thioether or thione groups containing various heterocyclic fragments with good yields of 42–80%. The interaction of 3,5-di-*tert*-butyl-*o*-benzoquinone with heterocyclic thiols leads to the formation of S-functionalized catechols. In the case of the acid-catalyzed reaction between 3,5-di-*tert*-butyl-6-methoxymethylcatechol and mercapto-substituted thiazole or pyridine derivatives, catechol−thioethers are formed with an additional methylene bridge between the catechol ring and the sulfur atom. In contrast, the mercapto derivatives of 1,3,4-oxadiazole or 1,2,4-triazole undergo alkylation at the nitrogen atom in the reaction with 3,5-di-*tert*-butyl-6-methoxymethylcatechol which leads to the corresponding catechol thiones. The molecular structures of catechols with a 2-pyridine or 1,3,4-oxadiazole-2-thione ring in the crystalline state were studied by X-ray diffraction.

It has been established that the number of heteroatoms in the cycle which are bonded to the catechol ring through a sulfur atom and/or methylene bridge as well as the presence of thioether or thione groups affect the electrochemical behavior of catechols. The electrochemical data made it possible to determine compounds characterized by minimal anodic potentials (**4** and **9**) which may exhibit more pronounced antioxidant activity among the studied compounds.

Catechol thioethers **3** and **4** proved to be the most effective scavengers of the DPPH radical. Thiones **6**, **7**, and **9** showed pronounced antiradical activity in the reaction with ABTS^·+^. Compounds **3**, **4**, and **8** showed high inhibitory activity in NBT-test. All studied compounds have antioxidant effects on the process of lipid peroxidation. Thus, in most of the studied assays, compounds **3** and **4** with a 4-phenylthiazole moiety turned out to be effective antioxidants among catechol thioethers, and **8** and **9** among thiones with a catechol fragment, which is consistent with electrochemical data.

## Supporting Information

File 1 Experimental procedures and characterization data.

## Data Availability

All data that supports the findings of this study is available in the published article and/or the supporting information to this article.
