# Supplementary material for: Synthesis, electrochemical properties, and antioxidant activity of sterically hindered catechols with 1,3,4-oxadiazole, 1,2,4-triazole, thiazole or pyridine fragments
Source: Beilstein J Org Chem. 2024 Sep 19;20:2378–91. doi: 10.3762/bjoc.20.202 (PMC11420547; doi:10.3762/bjoc.20.202)
Supplement: File 1 — Experimental procedures and characterization data. [file Beilstein_J_Org_Chem-20-2378-s001.pdf]

BEILSTEIN JOURNAL OF ORGANIC CHEMISTRY

Supporting Information

for

Synthesis, electrochemical properties, and antioxidant activity of sterically hindered catechols with 1,3,4-oxadiazole, 1,2,4-triazole, thiazole or pyridine fragments

Daria A. Burmistrova, Andrey Galustyan, Nadezhda P. Pomortseva, Kristina D. Pashaeva, Maxim V. Arsenyev, Oleg P. Demidov, Mikhail A. Kiskin, Andrey I. Poddel’sky, Nadezhda T. Berberova and Ivan V. Smolyaninov

Beilstein J. Org. Chem. **2024,** *20,* 2378–2391. doi:10.3762/bjoc.20.202

Experimental procedures and characterization data

License and Terms: This is a supporting information file under the terms of the Creative Commons Attribution License (https://creativecommons.org/licenses/by/4.0). Please note that the reuse, redistribution and reproduction in particular requires that the author(s) and source are credited and that individual graphics may be subject to special legal provisions.
The license is subject to the *Beilstein Journal of Organic Chemistry* terms and conditions: (https://www.beilstein-journals.org/bjoc/terms)

Content

S1. Experimental procedures 4

S1.1. General 4

S1.2. Instrumentation 4

S1.3. Synthesis and characterization 5

S1.4. X-Ray structures 12

S1.5. Antioxidant activity assay 14

S1.5.1. DPPH radical scavenging activity assay 14

S1.5.2. ABTS assay 15

S1.5.3. CUPRAC assay 15

S1.5.4. Inhibition of superoxide radical anion formation by xanthine oxidase (NBT assay) 16

S1.5.5. Lipid peroxidation of rat liver homogenate 16

S2. NMR-Spectra 17

Figure S1. The ^{1}H NMR spectrum of 4,6-di-*tert*-butyl-3-(5-*p*-tolyl-1,3,4-oxadiazol-2-ylthio)benzene-1,2-diol (1) in DMSO-d_6. 17

Figure S2. The $^{13}C\{^{1}H\}$ NMR spectrum of 4,6-di-*tert*-butyl-3-(5-*p*-tolyl-1,3,4-oxadiazol-2-ylthio)benzene-1,2-diol (**1**) in DMSO-d_6. 17

Figure S3. The ^{1}H NMR spectrum of 4,6-di-*tert*-butyl-3-(5-(4-chlorophenyl)-1,3,4-oxadiazol-2-ylthio)benzene-1,2-diol (**2**) in $CDCl_3$. 18

Figure S4. The $^{13}C\{^{1}H\}$ NMR spectrum of 4,6-di-*tert*-butyl-3-(5-(4-chlorophenyl)-1,3,4-oxadiazol-2-ylthio)benzene-1,2-diol (**2**) in $CDCl_3$. 18

Figure S5. The ^{1}H NMR spectrum of 4,6-di-*tert*-butyl-3-(4-phenylthiazol-2-ylthio)-benzene-1,2-diol (**3**) in $CDCl_3$. 19

Figure S6. The $^{13}C\{^{1}H\}$ NMR spectrum of 4,6-di-*tert*-butyl-3-(4-phenylthiazol-2-ylthio)-benzene-1,2-diol (**3**) in $CDCl_3$. 19

Figure S7. The ^{1}H NMR spectrum of 4,6-di-*tert*-butyl-3-((4-phenylthiazol-2-ylthio)methyl)benzene-1,2-diol (**4**) in $CDCl_3$ 20

Figure S8. The $^{13}C\{^{1}H\}$ NMR spectrum of 4,6-di-*tert*-butyl-3-((4-phenylthiazol-2-ylthio)methyl)benzene-1,2-diol (**4**) in $CDCl_3$. 20

Figure S9. The ^{1}H NMR spectrum of 4,6-di-*tert*-butyl-3-((3-nitropyridin-2-ylthio)methyl)benzene-1,2-diol (**5**) in $CDCl_3$ 21

Figure S10. The ^{13}C{^{1}H} NMR spectrum of 4,6-di-*tert*-butyl-3-((3-nitropyridin-2-ylthio)methyl)benzene-1,2-diol (**5**) in $CDCl_3$. 21

Figure S11. The ^{1}H NMR spectrum of 3-(4,6-di-*tert*-butyl-2,3-dihydroxybenzyl)-5-*p*-tolyl-1,3,4-oxadiazole-2(3*H*)-thione (**6**) in $CDCl_3$. 22

Figure S12. The ^{13}C{^{1}H} NMR spectrum of 3-(4,6-di-*tert*-butyl-2,3-dihydroxybenzyl)-5-*p*-tolyl-1,3,4-oxadiazole-2(3*H*)-thione (**6**) in $CDCl_3$. 22

Figure S13. The ^{1}H NMR spectrum of 5-(4-chlorophenyl)-3-(4,6-di-*tert*-butyl-2,3-dihydroxybenzyl)-1,3,4-oxadiazole-2(3*H*)-thione (**7**) in $CDCl_3$. 23

Figure S14. The ^{13}C{^{1}H} NMR spectrum of 5-(4-chlorophenyl)-3-(4,6-di-*tert*-butyl-2,3-dihydroxybenzyl)-1,3,4-oxadiazole-2(3*H*)-thione (**7**) in $CDCl_3$. 23

Figure S15. The ^{1}H NMR spectrum of 3-(4,6-di-*tert*-butyl-2,3-dihydroxybenzyl)-5-(pyridin-3-yl)-1,3,4-oxadiazole-2(3*H*)-thione (**8**) in $CDCl_3$. 24

Figure S16. The ^{13}C{^{1}H} NMR spectrum of 3-(4,6-di-*tert*-butyl-2,3-dihydroxybenzyl)-5-(pyridin-3-yl)-1,3,4-oxadiazole-2(3*H*)-thione (**8**) in $CDCl_3$. 24

Figure S17. The ^{1}H NMR spectrum of 1-(4,6-di-*tert*-butyl-2,3-dihydroxybenzyl)-3-(pyridin-3-yl)-1*H*-1,2,4-triazole-5(4*H*)-thione (**9**) in DMSO-d_6. 25

Figure S18. The ^{13}C{^{1}H} NMR spectrum of 1-(4,6-di-*tert*-butyl-2,3-dihydroxybenzyl)-3-(pyridin-3-yl)-1*H*-1,2,4-triazole-5(4*H*)-thione (**9**) in DMSO-d_6. 25

S3. Electrochemical data 26

Figure S19. The CV curve of **1** 26

Figure S20. The CV curve of **4** 26

Figure S21. The CV curve of **5** 27

Figure S22. The CV curve of **6** 27

Figure S23. The CV curve of **8** 28

Figure S24. The CV curve of **9** 28

S4. References 29

S1. Experimental procedures

S1.1. General

All starting reagents were commercially available: 3,5-di-*tert*-butyl-*o*-benzoquinone (99%, Alfa Aesar), 2-mercapto-4-phenylthiazole (98%, Alfa Aesar), 5-(4-chlorophenyl)-1,3,4-oxadiazole-2-thiol (97%, Aldrich), 5-(3-pyridyl)-1,3,4-oxadiazole-2-thiol (97%, Aldrich), 5-(3-pyridyl)-4*H*-triazole-3-thiol (97%, Aldrich), 5-(4-methylphenyl)-1,3,4-oxadiazole-2-thiol (97%, Aldrich), 3-nitropyridine-2-thiol (98%, TCI), 2,2'-azobis(2-amidinopropane) dihydrochloride (AAPH) (Aldrich, 97%), 2,2'-azino-bis(3-ethylbenzothiazoline-6-sulfonic acid) (≥98%, TCI), neocuproine (Sigma-Aldrich, 98%) 2,2-diphenyl-1-picrylhydrazyl (Aldrich), thiobarbituric acid (≥98%, Sigma-Aldrich), deoxyribonucleic acid sodium salt (DNA) from salmon testes (Sigma), phosphate buffered saline (PBS) pH 7.4 (Sigma), xanthine oxidase from bovine (Sigma-Aldrich, grade IV), nitroblue tetrazolium (90%, Alfa Aesar), xanthine (≥99% Sigma-Aldrich), bovine serum albumin (≥96%, Sigma-Aldrich). 3,5-Di-*tert*-butyl-6-methoxymethylcatechol was synthesized by a known method [1]. Standard procedures have been used to purify solvents [2].

S1.2. Instrumentation

An FSM-1201 FT-IR spectrometer was used to record IR spectra in KBr pellets. The ^{1}H and $^{13}C\{^{1}H\}$ NMR spectra were measured in $CDCl_3$ on a Bruker Avance HD 400 spectrometer with a frequency of 400 MHz for ^{1}H and 100 MHz for $^{13}C\{1H\}$ NMR spectra. The chemical shift values are given in ppm with reference to the solvent, and the coupling constants (J) are given in Hz. The Euro EA 3000 (C,H,N) elemental analyzer was used to determine the elemental composition of the synthesized substances. High-resolution mass spectra (HRMS) were recorded on a mass spectrometer Bruker UHR-TOF Maxis™ (ESI).

To determine the electrochemical potentials of catechols **1–9** and starting thiols, a technique of cyclic voltammetry (CV) was used. The measurements occurred in a three-

electrode cell on a VersaSTAT3 (Princeton Applied Research) potentiostate in CH_3CN under argon. A stationary glassy carbon (GC) electrode with a diameter of 2 mm was the working electrode and a platinum plate (S = 18 mm^2) served as the auxiliary electrode. The potentials of the complexes were measured versus the reference electrode (Ag/AgCl/KCl) with a waterproof membrane. The number of electrons transferred during the electrode process was estimated relative to ferrocene as the standard. The concentration of compounds was 1–3 mmol.

Microelectrolysis of catechols **3** and **6** was performed on a VersaSTAT3 potentiostate at stationary platinum electrodes (S = 18 mm^2) in an undivided three-electrode cell (2 mL) under anaerobic conditions. Catechol was added to an electrochemical cell containing a solution of the supporting electrolyte (0.1 M Bu_4NClO_4) in acetonitrile. Electrolysis was performed in the potentiostatic mode at a potential of 1.30–1.35 V.

S1.3. Synthesis and characterization

Catechol thioethers **1–3** were obtained from 3,5-di-*tert*-butyl-*o*-benzoquinone (0.44 g, 2 mmol) and corresponding thiol (2 mmol) in ethanol under an inert atmosphere (argon) based on the known method [3]. Compounds **4–9** were synthesized in the reaction of 3,5-di-*tert*-butyl-6-methoxymethylcatechol (0.54 g, 2 mmol) with thiols (2 mmol) in chloroform (**4**, **6–9**) or acetic acid (**5**) (10 mL) under argon with stirring for 6 h at 60 °C. The solvent was evaporated under reduced pressure; the formed precipitate was recrystallized from acetonitrile. In the case of **5**, after the reaction, 20 mL of water was added to the solution; the resulting precipitate was filtered off, dried in a vacuum, and recrystallized from acetonitrile.

4,6-Di-*tert*-butyl-3-(5-*p*-tolyl-1,3,4-oxadiazol-2-ylthio)benzene-1,2-diol (1)

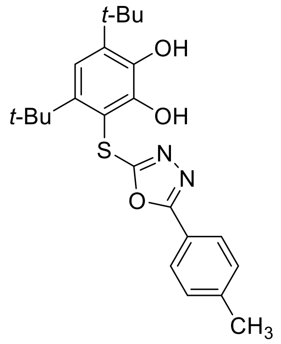

Yield 0.570 g (69%). White powder with m.p. 300–302 ºC.

IR (KBr, ν/cm^{-1}): 3438, 3218, 2954, 2907, 2870, 1675, 1624, 1571, 1541, 1499, 1465, 1422, 1401, 1365, 1338, 1307, 1287, 1244, 1206, 1188, 1165, 1130, 1030.

^{1}H NMR (400 MHz, DMSO-d$_6$, δ, ppm): 1.31 (s, 9H, tBu), 1.38 (s, 9H, tBu), 2.39 (s, 3H, CH_3), 7.02 (s., 1H, arom. C_6H_1), 7.35 (d., 3J(H,H) = 8.0 Hz, 2H, arom. C_6H_4), 7.77 (d., 3J(H,H) = 8.0 Hz, 2H, arom. C_6H_4), 9.58 (br.s., 1H, OH), 10.41 (br.s., 1H, OH).

^{13}C{^{1}H} NMR (100 MHz, DMSO-d$_6$, δ, ppm): 21.10, 29.25, 29.26, 34.55, 34.98, 116.01, 119.52, 127.89, 129.14, 130.57, 133.67, 134.72, 138.39, 139.97, 141.75, 152.30, 162.21.

HR-MS: Found m/z: 435.1696 [M+Na]$^+$. $C_{23}H_{28}N_2NaO_3S$. Calcd. m/z: 435.1713.

4,6-Di-*tert*-butyl-3-(5-(4-chlorophenyl)-1,3,4-oxadiazol-2-ylthio)benzene-1,2-diol (2)

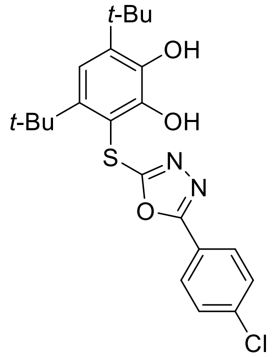

Yield 0.690 g (80%). White powder with m.p. 299–301 ºC.

IR (KBr, ν/cm^{-1}): 3438, 3286, 2958, 2907, 2870, 1671, 1638, 1620, 1567, 1534, 1498, 1485, 1464, 1400, 1364, 1400, 1364, 1307, 1277, 1240, 1174, 1163, 1126, 1027.

^{1}H NMR (400 MHz, $CDCl_3$, δ, ppm): 1.32 (s, 9H, tBu), 1.38 (s, 9H, tBu), 7.04 (s, 1H, arom. C_6H_1), 7.43 (d., ^3J(H,H)=8.6, 2H, arom. C_6H_4), 7.90 (d., ^3J(H,H)=8.6, 2H, arom. C_6H_4), 9.06 (s., 1H, OH), 11.56 (s., 1H, OH).

$^{13}C\{^1H\}$ NMR (100 MHz, $CDCl_3$, δ, ppm): 29.31, 29.61, 34.84, 35.18, 116.11, 119.98, 127.89, 130.31, 131.70, 134.17, 135.28, 137.69, 138.76, 139.82, 152.79, 170.49.

HR-MS: Found m/z: 455.1173 $[M+Na]^+$. $C_{22}H_{25}ClN_2NaO_3S$. Calcd. m/z: 455.1167.

4,6-Di-*tert*-butyl-3-(4-phenylthiazol-2-ylthio)benzene-1,2-diol (3)

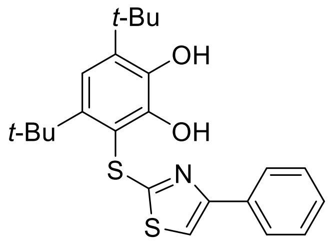

Yield 0.621 g (75%). Red-brown powder with m.p. 142–144 ºC.

IR (KBr, ν/cm^{-1}): 3482, 3113, 2958, 2907, 2870, 1602, 1559, 1481, 1444, 1418, 1397, 1372, 1360, 1292, 1262, 1242, 1220, 1170, 1058, 1026.

^{1}H NMR (400 MHz, $CDCl_3$, δ, ppm): 1.42 (s, 9H, tBu), 1.51 (s, 9H, tBu), 6.28 (br.s, 1H, OH), 7.01 (s., 1H, arom. C_6H_1), 7.33-7.50 (m., 5H: 3H, arom. C_6H_5 + 1H, C_3HNS + 1H, OH), 7.80 (d.m., ^3J(H,H) = 7.9 Hz, 2H, arom. C_6H_5).

$^{13}C\{^1H\}$ NMR (100 MHz, $CDCl_3$, δ, ppm): 29.18, 31.63, 35.19, 36.93, 110.41, 114.38, 115.12, 117.25, 126.18, 128.97, 132.52, 137.09, 141.99, 143.76, 146.29, 155.23, 166.07.

HR-MS: Found m/z: 414.1553 $[M+H]^+$. $C_{23}H_{28}NO_2S_2$. Calcd. m/z: 414.1556.

4,6-Di-*tert*-butyl-3-((4-phenylthiazol-2-ylthio)methyl)benzene-1,2-diol (4)

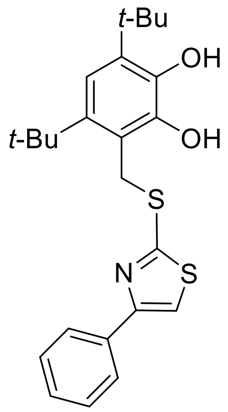

Yield 0.685 g (80%). Beige powder with m.p. 152–154 °C.

IR (KBr, ν/cm^{-1}): 3481, 3105, 2967, 2911, 2870, 1508, 1485, 1471, 1421, 1396, 1379, 1356, 1288, 1268, 1249, 1221, 1201, 1185, 1172, 1054, 1044.

^{1}H NMR (400 MHz, $CDCl_3$, δ, ppm): 1.41 (s, 9H, tBu), 1.44 (s, 9H, tBu), 4.91 (s., 2H, CH_2), 6.37 (br.s, 1H, OH), 6.93 (s., 1H, arom. C_6H_1), 7.35 (s., 1H, C_3HNS), 7.40-7.44 (m., 1H, arom. C_6H_5), 7.46-7.51 (m., 2H, arom. C_6H_5), 7.88 (d.m., ^{3}J(H,H) = 8.0 Hz, 2H, arom. C_6H_5).

$^{13}C\{^{1}H\}$ NMR (100 MHz, $CDCl_3$, δ, ppm): 29.33, 32.66, 34.49, 34.87, 35.77, 113.29, 116.12, 120.34, 126.34, 128.85, 129.03, 132.69, 133.73, 137.87, 143.05, 143.67, 154.42, 168.85.

HR-MS: Found m/z: 428.1724 $[M+H]^+$. $C_{24}H_{30}NO_2S_2$. Calcd. m/z: 428.1712.

4,6-Di-*tert*-butyl-3-((3-nitropyridin-2-ylthio)methyl)benzene-1,2-diol (5)

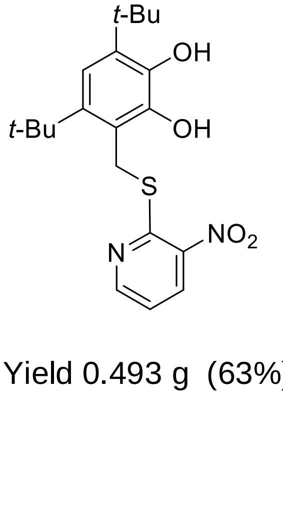

Yield 0.493 g (63%). Orange crystals with m.p. 143–145 °C.

IR (KBr, ν/cm^{-1}): 3503, 3126, 3075, 2961, 2955, 2910, 2872, 1592, 1558, 1520, 1483, 1430, 1402, 1326, 1360, 1290, 1269, 1254, 1234, 1216, 1171, 1125.

^{1}H NMR (400 MHz, $CDCl_3$, δ, ppm): 1.41 (s, 9H, tBu), 1.48 (s, 9H, tBu), 4.80 (s, 2H, CH_2), 6.09 (s, 1H, OH), 6.95 (s, 1H, arom. C_6H_1), 7.33 (dd, J = 8.2 and 4.8 Hz, 1H, o-Py), 8.55 (dd, J = 8.2 and 1.6 Hz, 1H, o-Py), 8.76 (dd, J = 4.8 and 1.6 Hz, 1H, o-Py), 9.84 (s, 1H, OH).

^{13}C{^{1}H} NMR (100 MHz, $CDCl_3$, δ, ppm): 29.35, 30.34, 32.80, 34.84, 35.82, 116.28, 118.94, 119.60, 133.71, 134.88, 138.52, 142.28, 142.77, 143.61, 152.13, 158.63.

Calcd. for $C_{20}H_{26}N_2O_4S$ (%): C, 61.52; H, 6.71; N, 7.17 Found (%): C, 61.43; H, 6.92; N, 7.25.

3-(4,6-Di-*tert*-butyl-2,3-dihydroxybenzyl)-5-*p*-tolyl-1,3,4-oxadiazole-2(3*H*)-thione (6)

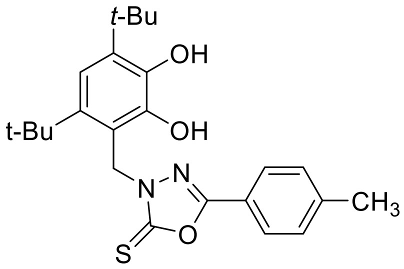

Yield 0.675 g (79%). Beige crystals with m.p. 128–130 ^{o}C.

IR (KBr, ν/cm^{-1}): 3506, 3487, 3276, 2965, 2955, 2909, 2871, 1619, 1605, 1570, 1560, 1542, 1508, 1456, 1420, 1373, 1360, 1335, 1294, 1274, 1245, 1215, 1182, 1164, 1109, 1078.

^{1}H NMR (400 MHz, $CDCl_3$, δ, ppm): 1.42 (s, 9H, tBu), 1.49 (s, 9H, tBu), 2.42 (s, 3H, CH_3), 5.60 (s, 2H, CH_2), 6.16 (br.s, 1H, OH), 7.05 (s, 1H, arom. C_6H_1), 7.11 (br.s, 1H, OH), 7.29 (d., ^{3}J(H,H)=8.2, 2H, arom. C_6H_4), 7.76 (d., ^{3}J(H,H)=8.2, 2H, arom. C_6H_4).

^{13}C{^{1}H} NMR (100 MHz, $CDCl_3$, δ, ppm): 21.75, 29.29, 32.50, 35.02, 35.56, 46.45, 117.02, 117.73, 118.81, 126.64, 129.92, 135.41, 139.86, 143.41, 143.56, 143.85, 160.08, 175.75.

HR-MS: Found m/z: 449.1865 $[M+Na]^+$. $C_{24}H_{30}N_2NaO_3S$. Calcd. m/z: 449.1869.

5-(4-Chlorophenyl)-3-(4,6-di-*tert*-butyl-2,3-dihydroxybenzyl)-1,3,4-oxadiazole-2(3*H*)-thione (7)

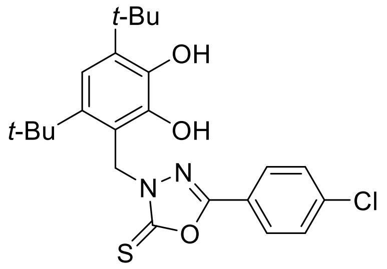

Yield 0.358 g (40%). Beige powder with m.p. 158–160 ºC.

IR (KBr, ν/cm^{-1}): 3491, 3367, 2963, 2911, 2872, 1616, 1595, 1565, 1491, 1422, 1407, 1371, 1357, 1342, 1285, 1268, 1243, 1222, 1179, 1169, 1115, 1091, 1092, 1044, 1027.

^{1}H NMR (400 MHz, $CDCl_3$, δ, ppm): 1.42 (s, 9H, tBu), 1.49 (s, 9H, tBu), 5.61 (s, 2H, CH_2), 6.06 (s, 1H, OH), 6.83 (s, 1H, OH), 7.05 (s, 1H, arom. C_6H_1), 7.47 (d., ^{3}J(H,H)=8.5, 2H, arom. C_6H_4), 7.81 (d., ^{3}J(H,H)=8.5, 2H, arom. C_6H_4).

^{13}C{^{1}H} NMR (100 MHz, $CDCl_3$, δ, ppm): 29.32, 32.53, 35.01, 35.58, 46.58, 116.67, 117.66, 120.19, 127.90, 129.67, 135.51, 139.29, 139.88, 142.13, 143.59, 158.98, 175.97.

HR-MS: Found m/z: 469.1149 $[M+Na]^+$. $C_{23}H_{27}ClN_2NaO_3S$. Calcd. m/z: 469.1167.

3-(4,6-Di-*tert*-butyl-2,3-dihydroxybenzyl)-5-(pyridin-3-yl)-1,3,4-oxadiazole-2(3*H*)-thione (8)

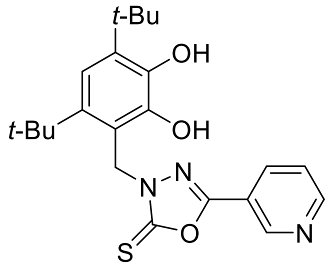

Yield 0.348 g (42%). Beige crystals with m.p. 198–200 ºC.

IR (KBr, ν/cm^{-1}): 3354, 2958, 2907, 2873, 2575, 1616, 1598, 1568, 1492, 1476, 1458, 1438, 1417, 1346, 1320, 1274, 1235, 1197, 1176, 1126, 1107, 1072, 1016.

^{1}H NMR (400 MHz, $CDCl_3$, δ, ppm): 1.41 (s, 9H, tBu), 1.48 (s, 9H, tBu), 5.63 (s, 2H, CH_2), 6.05 (br.s, 2H, OH), 7.05 (s, 1H, arom. C_6H_1), 7.43 (d.d., J = 4.9 and 7.9 Hz, 1H, m-Py), 8.12 (d.t, J = 7.9 and 1.7 Hz, 1H, m-Py), 8.75 (d.d., J = 4.9 and 1.3 Hz, 1H, m-Py), 9.05 (d, J = 1.7 Hz, 1H, m-Py).

$^{13}C\{^{1}H\}$ NMR (100 MHz, $CDCl_3$, δ, ppm): 29.37, 32.53, 35.01, 35.59, 46.77, 116.67, 117.58, 118.70, 123.88, 133.86, 135.68, 139.96, 142.97, 143.77, 147.41, 152.91, 157.44, 176.12.

HR-MS: Found m/z: 436.1654 $[M+Na]^+$. $C_{22}H_{27}N_3NaO_3S$. Calcd. m/z: 436.1665.

1-(4,6-Di-*tert*-butyl-2,3-dihydroxybenzyl)-3-(pyridin-3-yl)-1*H*-1,2,4-triazole-5(4*H*)-thione (9)

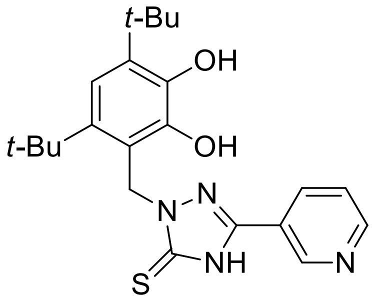

Yield 0.363 g (44%). White powder with m.p. 286–288 °C.

IR (KBr, ν/cm^{-1}): 3445, 3117, 3022, 2992, 2954, 2910, 2870, 1603, 1583, 1567, 1560, 1506, 1484, 1475, 1429, 1414, 1395, 1381, 1360, 1328, 1291, 1272, 1250, 1221, 1197, 1174, 1157, 1126, 1087.

^{1}H NMR (400 MHz, DMSO-d_6, δ, ppm): 1.34 (s, 9H, tBu), 1.37 (s, 9H, tBu), 4.74 (s, 2H, CH_2), 6.79 (s, 1H, arom. C_6H_1), 7.55 (d.d., J = 4.9 and 7.9 Hz, 1H, m-Py), 8.02 (br.s, 1H, OH), 8.32 (d.t, J = 7.9 and 1.8 Hz, 1H, m-Py), 8.6 (br.s, 1H, NH), 8.66 (d.d., J = 4.9 and 1.2 Hz, 1H, m-Py), 9.17 (d, J = 1.8 Hz, 1H, m-Py), 14.38 (br.s, 1H, OH).

$^{13}C\{^{1}H\}$ NMR (100 MHz, DMSO-d_6, δ, ppm): 29.52, 31.94, 34.67, 35.34, 115.32, 120.79, 124.12, 133.32, 135.04, 138.21, 142.92, 145.74, 146.68, 146.93, 150.47, 151.32, 172.10. (the signal of CH_2 group is covered by a DMSO solvent signal).

HR-MS: Found m/z: 413.1992 $[M+H]^+$. $C_{22}H_{29}N_4O_2S$. Calcd. m/z: 413.2006.

S1.4. X-ray structures

Crystals of compounds **5**, **6**, and **8** suitable for X-ray diffraction were obtained by slow evaporation of acetonitrile at room temperature. An experimental array of reflection was obtained by the standard method on a Bruker Smart APEX II diffractometer (Mo Kα radiation, λ = 0.71073 Å) (for **5**) or on an Agilent SuperNova diffractometer using a microfocus X-ray source with a copper anode and an Atlas S2 coordinate CCD detector (for **6** and **8**). Empirical absorption corrections were applied for **5** using SADABS program [4]. Reflections collection, determination and refinement of unit cell parameters for **6** and **8** was carried out using CrysAlisPro software (version 1.171.38.41) [5]. The structures were solved taking into account crystal twinning in OLEX2 or program [6,7] by using the Intrinsic Phasing method and refined with SHELXL [8,9] by the full-matrix least-squares method against F^2 with anisotropic thermal parameters for all non-hydrogen atoms. Non-hydrogen atoms were refined in anisotropic approximation. Hydrogen atoms of the carbon-containing ligands were geometrically generated and refined in the riding model. The structure of **5** was solved by taking into account the disordering 3-nitropyridin-2-ylthio fragment in two positions (0.602(16)/0.398(16)). Some restrictions were applied in structure solvation (EADP and ISOR **5**). The crystallographic data for the structures reported in this paper have been deposited with the Cambridge Crystallographic Data Center (2347998 for **5**, 2347786 for **6**·0.5CH_3CN, 2347798 for **8**). The crystallographic parameters and X-ray diffraction experimental parameters are given in Table S1.

Table S1: Selected crystal data for **5**, **6**·0.5CH3CN, and **8**.

**Compound**	**5**	**6**·0.5CH_3CN	**8**
CCDC Number	2347998	2347786	2347798
Empirical formula	$C_{20}H_{26}N_2O_4S$	$C_{50}H_{63}N_5O_6S_2$	$C_{22}H_{27}N_3O_3S$
Formula weight	390.49	894.17	413.52
Temperature/K	150(2)	293(2)	293(2)
Crystal system	orthorhombic	monoclinic	triclinic
Space group	Pnc2	$P2_1/c$	P-1
a/Å	17.2984(17)	22.2148(4)	9.41412(17)
b/Å	13.0751(13)	11.9176(2)	10.0263(2)
c/Å	8.7293(9)	18.6134(3)	12.8519(3)
α/°	90	90	73.338(2)
β/°	90	95.632(2)	71.170(2)
γ/°	90	90	81.3543(16)
Volume/Å^3	1974.4(3)	4904.06(15)	1097.70(5)
Z	4	4	2
ρ_{calc}g/cm^3	1.314	1.211	1.251
μ/mm^{-1}	0.192	1.400	1.530
F(000)	832	1912	440
Crystal size/mm^3	0.20 × 0.18 × 0.18	0.411 × 0.111 × 0.07	0.417 × 0.376 × 0.114
Radiation	MoKα (λ = 0.71073)	CuKα (λ = 1.54184)	CuKα (λ = 1.54184)
2Θ range for data collection/°	4.710 to 56.558	7.998 to 153.092	7.516 to 152.572

Index ranges	$-23 \leq h \leq 22$<br>$-17 \leq k \leq 17$<br>$-11 \leq l \leq 11$	$-27 \leq h \leq 27$<br>$-11 \leq k \leq 14$<br>$-23 \leq l \leq 23$	$-11 \leq h \leq 8$<br>$-12 \leq k \leq 12$<br>$-16 \leq l \leq 16$
Reflections collected	27915	53253	22882
Independent reflections	4893 [R_{int} = 0.0841, R_{sigma} = 0.0682]	10248 [R_{int} = 0.0307, R_{sigma} = 0.0200]	4582 [R_{int} = 0.0329, R_{sigma} = 0.0208]
Data/restraints/parameters	4893/55/269	10248/0/599	4582/0/276
GOOF (F^2)	1.041	1.022	1.092
R_1/wR_2 ($I>=2\sigma(I)$)	0.0913 / 0.2487	0.0393 / 0.1044	0.0577 / 0.1646
R_1/wR_2 [all data]	0.1627 / 0.3147	0.0479 / 0.1125	0.0591 / 0.1655
Largest diff. peak/hole / eÅ^{-3}	0.73 / -0.34	0.17/-0.29	0.44/-0.26

S1.5. Antioxidant activity assay

S1.5.1. DPPH radical scavenging activity assay

DPPH radical scavenging activity was performed according to the known method [10] with some modifications [11]. A CH_2Cl_2 solution (c_0 = 50 µmol) of the radical DPPH was prepared daily and protected from light. The decrease in absorbance was determined at 527 nm every minute during the first five minutes of the experiment and every next 5 min until the reaction reached a plateau at room temperature. The parameter EC_{50} is the concentration of an antioxidant necessary for decreasing the amount of DPPH radical by 50% of the initial value. To determine IC_{50}, the plot of the residual concentration of the stable radical vs molar ratio, expressed as the number of moles of the antioxidant per 1 mole of the DPPH, was constructed. The parameter (n_{DPPH}) is the number of molecules of converted DPPH radical per one molecule of the compound ($n_{DPPH} = C_0/(2 \times IC_{50})$, where c_0 is the initial concentration of radical). TEC_{50} is the time of achievement of an

equilibrium state at the antioxidant concentration equal to IC_{50}. The antiradical efficiency (AE) was determined with the equation AE = 1/(IC_{50} × TEC_{50}). All experiments were performed in triplicate at room temperature.

S1.5.2. ABTS assay

The radical cation $ABTS^{\cdot+}$ is generated by the oxidation of ABTS with the $K_2S_2O_8$. The reduction in the intensity of greenish coloration characteristic of this radical reflects the ability of the antioxidants to scavenge the radical cation [12]. The absorbance of ABTS radical cation solutions (λ = 734 nm) in the presence of compounds **1–9** concentrations (1–40 µM) and calculation of the IC_{50} values were carried out following a previously published method [13]. Ethanol was the solvent for all compounds except for catechol **9** (DMSO). The absorbance (A_i) reading was taken at room temperature exactly 1 min after initial mixing and up to 6 min. All measurements were carried out at least three times. Plots of absorbance versus concentration were prepared for test compounds and Trolox. Trolox equivalent antioxidant capacity (TEAC) values were measured by comparing the slopes of plots obtained for each compound to that of Trolox. The absorbance of the blank (40 µL ethanol (DMSO) and 40 µL of radical cation) assay was set as 100% radical. The IC_{50} values were calculated as the minimum concentration of each sample required to inhibit 50% of the ABTS radical.

S1.5.3. CUPRAC assay

Cu(II) ion reducing (CUPRAC) assay was carried out by a known method [14]. The solution of $CuCl_2$ (0.01 M) is prepared by dissolving in aqueous ammonium acetate buffer (1 M). Neocuproine (Nc) (7.5 mM) is dissolved in 96% ethanol. In a test tube, 0.5 mL each of Cu(II), Nc, and ammonium acetate buffer solutions were added. The catechol or Trolox solution in ethanol (or DMSO in the case of **9**) and ethanol (96%) were added to the initial mixture to make the final volume 2 ml. The concentration of compounds in test tubes ranged from 10 to 50 µM. Absorbance was measured at 450 nm on a spectrophotometer

Akvilon SF-104 (Russia) against a reagent blank 30 min later. The standard calibration curves of each compound were constructed by plotting absorbance versus molar concentration. Trolox was used as the standard antioxidant for calculating TEAC. TEAC coefficient for this assay was determined by relating the molar absorptivity, ε of the test samples to that of Trolox as follows: TEAC = $\varepsilon/\varepsilon_{Trolox}$ (ε_{Trolox} = $1.79 \cdot 10^4$ $L \cdot mol^{-1} \cdot cm^{-1}$).

S1.5.4. Inhibition of superoxide radical anion formation by xanthine oxidase (NBT assay)

To evaluate the radical scavenging activity of compounds, a reaction of compounds **1**, **3**, **4,** and **8** with $O_2^{\cdot-}$ generated in the xanthine/xanthine oxidase enzymatic system was used according to the previously described method [15]. Inhibition I (%) = [(1 − A_i/A_0) × 100%], where A_i is the absorbance in the presence of catechol at the end of the reaction (800 s), A_0 is the absorbance of the blank solution. The IC_{50} values were determined graphically using the dependence of inhibition values versus the concentration of the compound. All experiments were performed three times.

S1.5.5. Lipid peroxidation of rat liver homogenate

The level of lipid peroxidation (LP) of rat Wistar liver homogenates was estimated by using the thiobarbituric acid reactive substances (TBARS) assay [16]. Samples of rat Wistar liver were homogenized (1:10 w/v) in phosphate buffer, pH 7.4 using a homogenizer. The intensity of lipid peroxidation at 37 °C as a non-enzymatic process in the presence of ascorbic acid and $(NH_4)_2Fe(SO_4)$ was measured as previously described in a known method [13]. The concentration of test compounds was 0.1 mM. TBARS concentrations were determined after 3, 24, and 48 h incubation. The values are expressed as mean % ± SD.

S2. NMR-Spectra

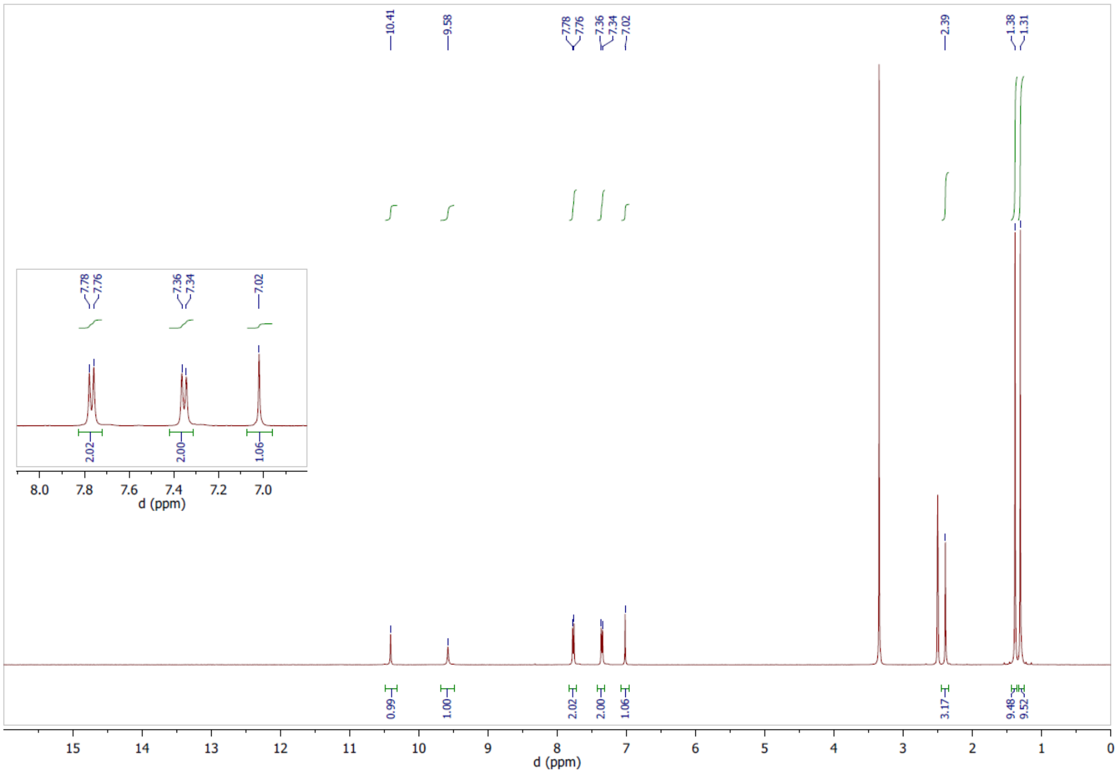

Figure S1. The ^{1}H NMR spectrum of 4,6-di-*tert*-butyl-3-(5-*p*-tolyl-1,3,4-oxadiazol-2-ylthio)benzene-1,2-diol (**1**) (400 MHz, DMSO-d_6).

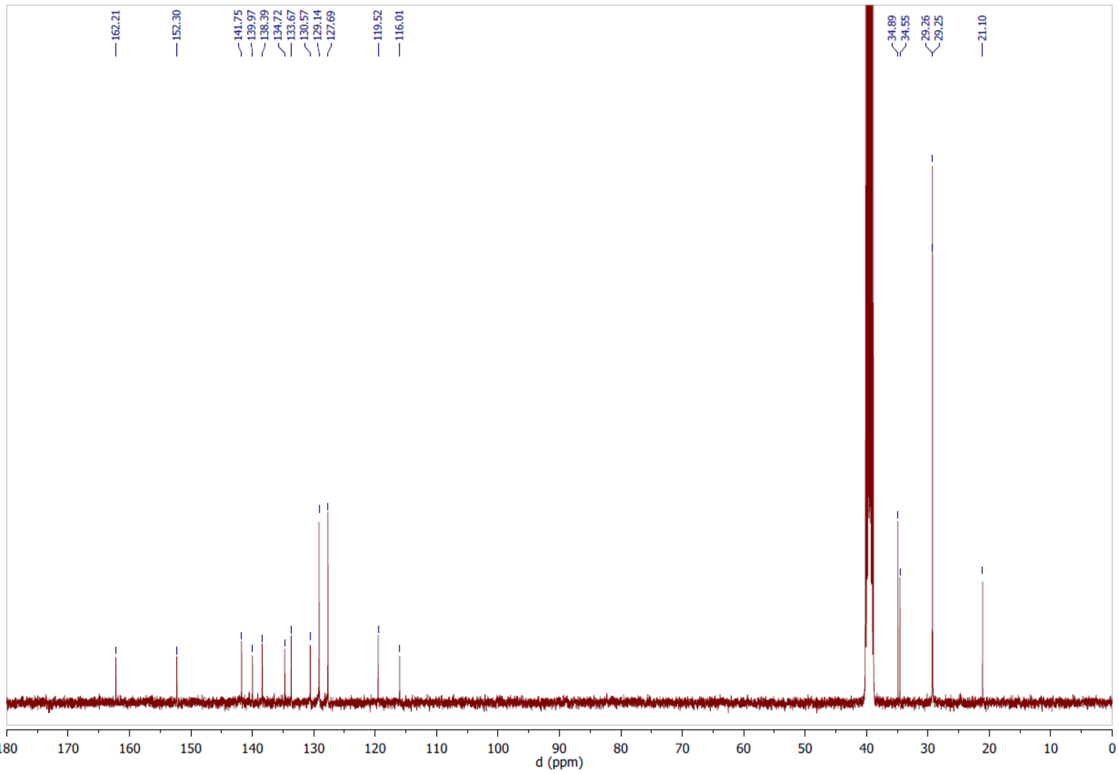

Figure S2. The ^{13}C{^{1}H} NMR spectrum of 4,6-di-*tert*-butyl-3-(5-*p*-tolyl-1,3,4-oxadiazol-2-ylthio)benzene-1,2-diol (**1**) (100 MHz, DMSO-d_6).

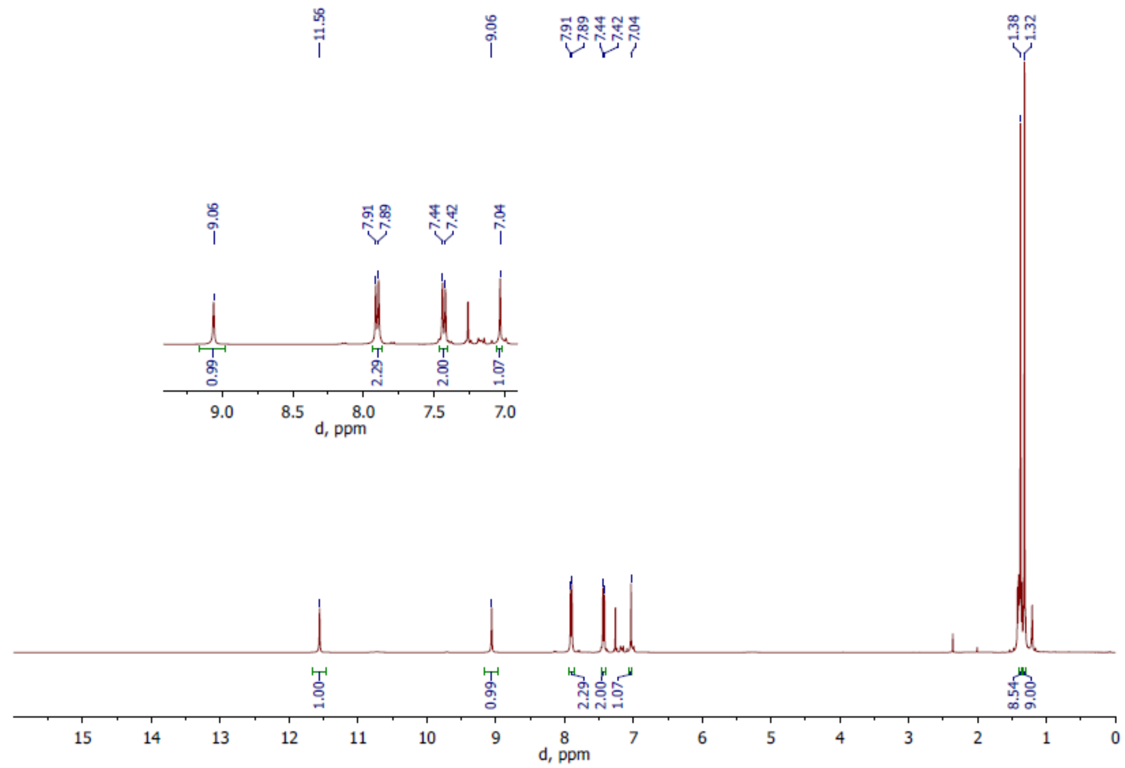

Figure S3. The ^{1}H NMR spectrum of 4,6-Di-*tert*-butyl-3-(5-(4-chlorophenyl)-1,3,4-oxadiazol-2-ylthio)benzene-1,2-diol (**2**) (400 MHz, $CDCl_3$).

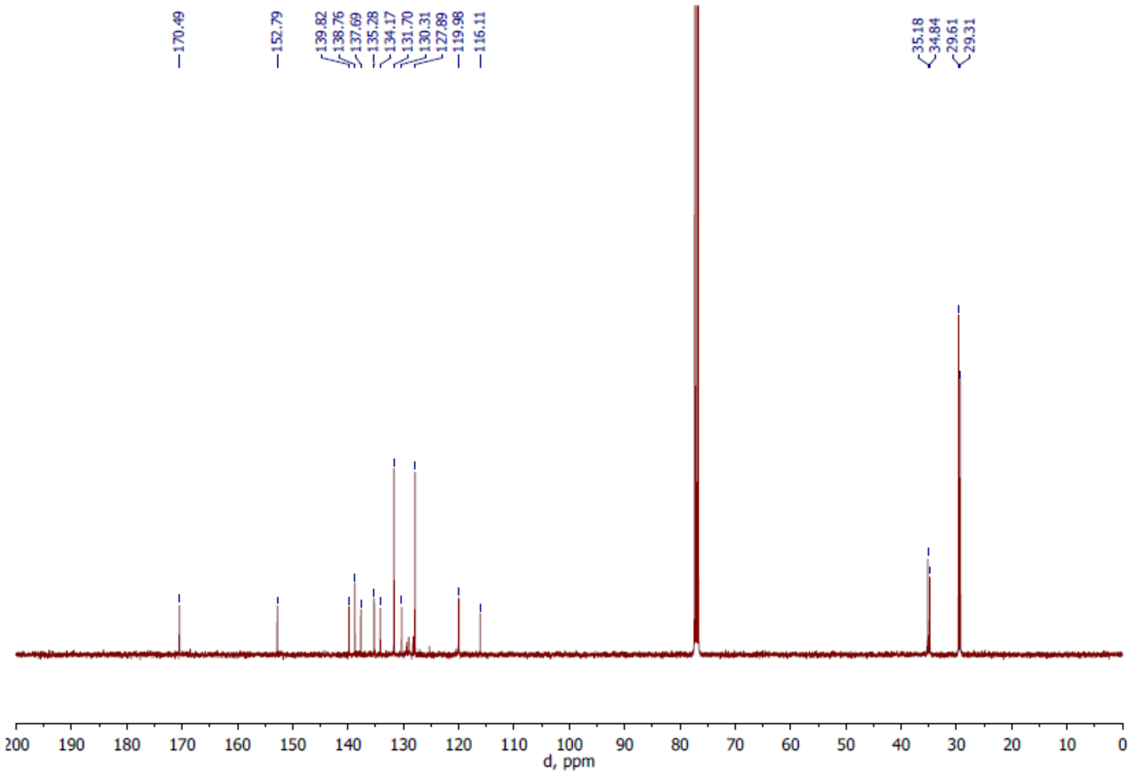

Figure S4. The $^{13}C\{^{1}H\}$ NMR spectrum of 4,6-Di-*tert*-butyl-3-(5-(4-chlorophenyl)-1,3,4-oxadiazol-2-ylthio)benzene-1,2-diol (**2**) (100 MHz, $CDCl_3$).

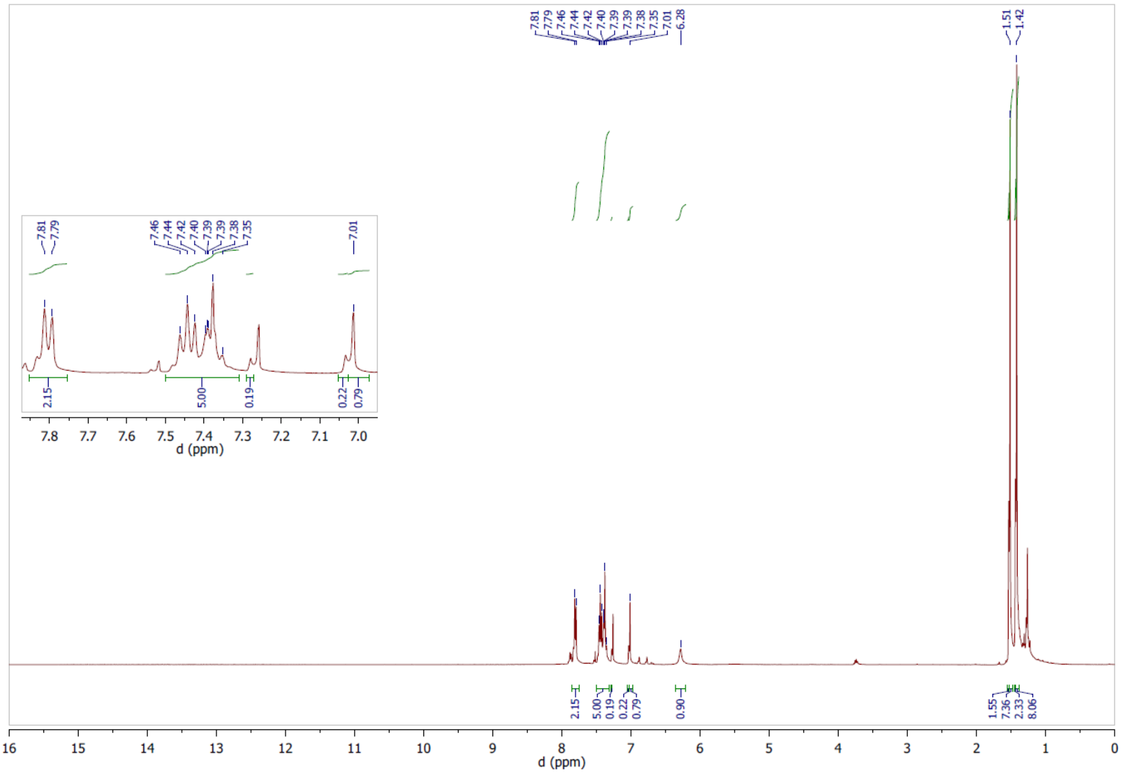

Figure S5. The ^{1}H NMR spectrum of 4,6-di-*tert*-butyl-3-(4-phenylthiazol-2-ylthio)benzene-1,2-diol (**3**) (400 MHz, $CDCl_3$).

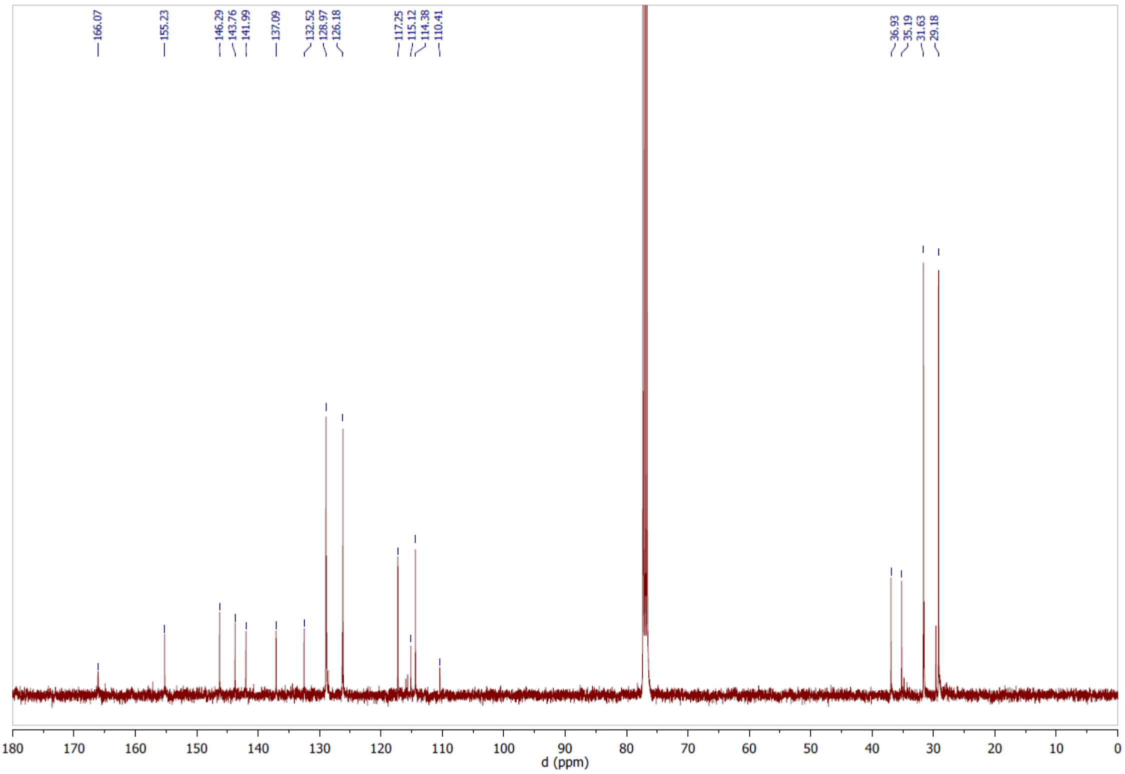

Figure S6. The $^{13}C\{^{1}H\}$ NMR spectrum of 4,6-di-*tert*-butyl-3-(4-phenylthiazol-2-ylthio)benzene-1,2-diol (**3**) (100 MHz, $CDCl_3$).

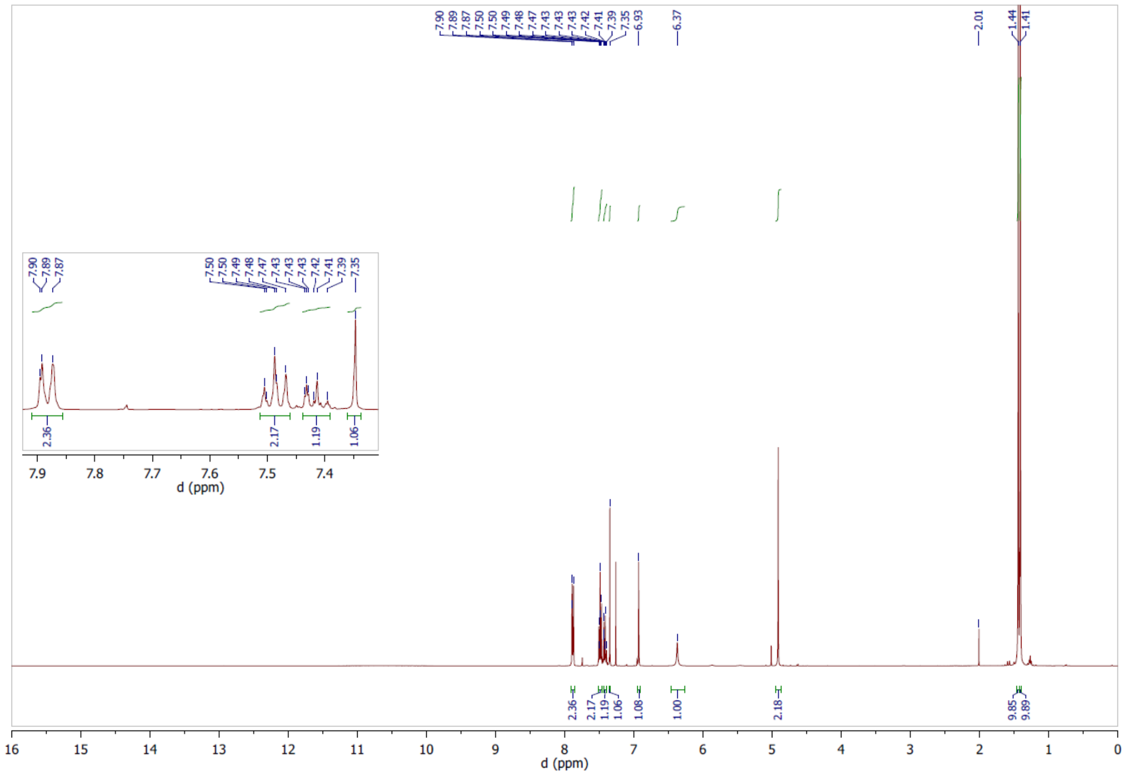

Figure S7. The ^{1}H NMR spectrum of 4,6-di-*tert*-butyl-3-((4-phenylthiazol-2-ylthio)methyl)benzene-1,2-diol (**4**) (400 MHz, $CDCl_3$).

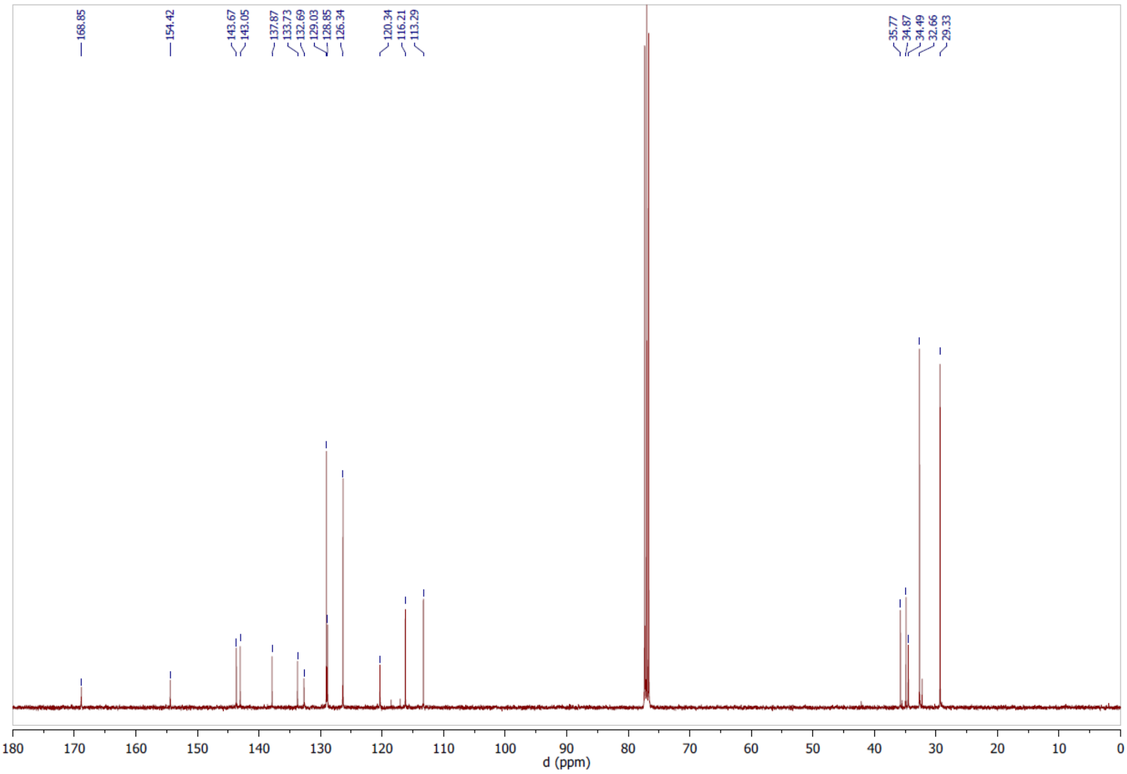

Figure S8. The $^{13}C\{^{1}H\}$ NMR spectrum of 4,6-di-*tert*-butyl-3-((4-phenylthiazol-2-ylthio)methyl)benzene-1,2-diol (**4**) (100 MHz, $CDCl_3$).

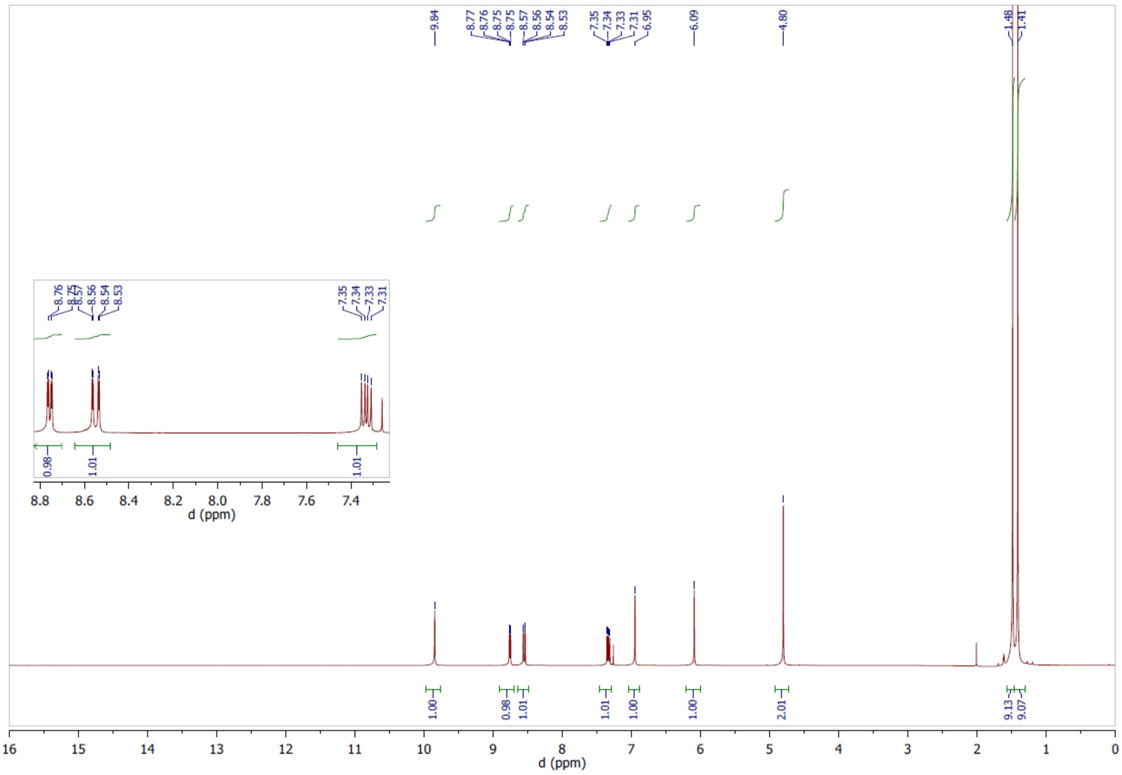

Figure S9. The ¹H NMR spectrum of 4,6-di-*tert*-butyl-3-((3-nitropyridin-2-ylthio)methyl)benzene-1,2-diol (**5**) (400 MHz, $CDCl_3$).

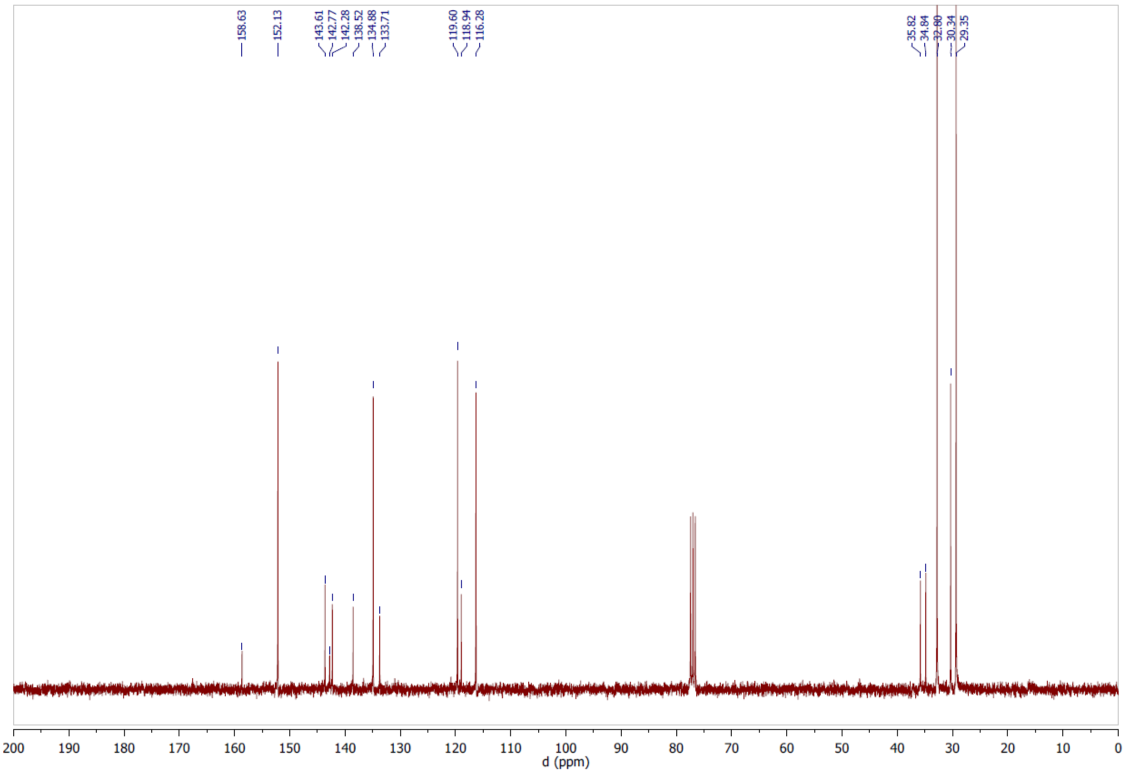

Figure S10. The $^{13}C\{^1H\}$ NMR spectrum of 4,6-di-*tert*-butyl-3-((3-nitropyridin-2-ylthio)methyl)benzene-1,2-diol (**5**) (100 MHz, $CDCl_3$).

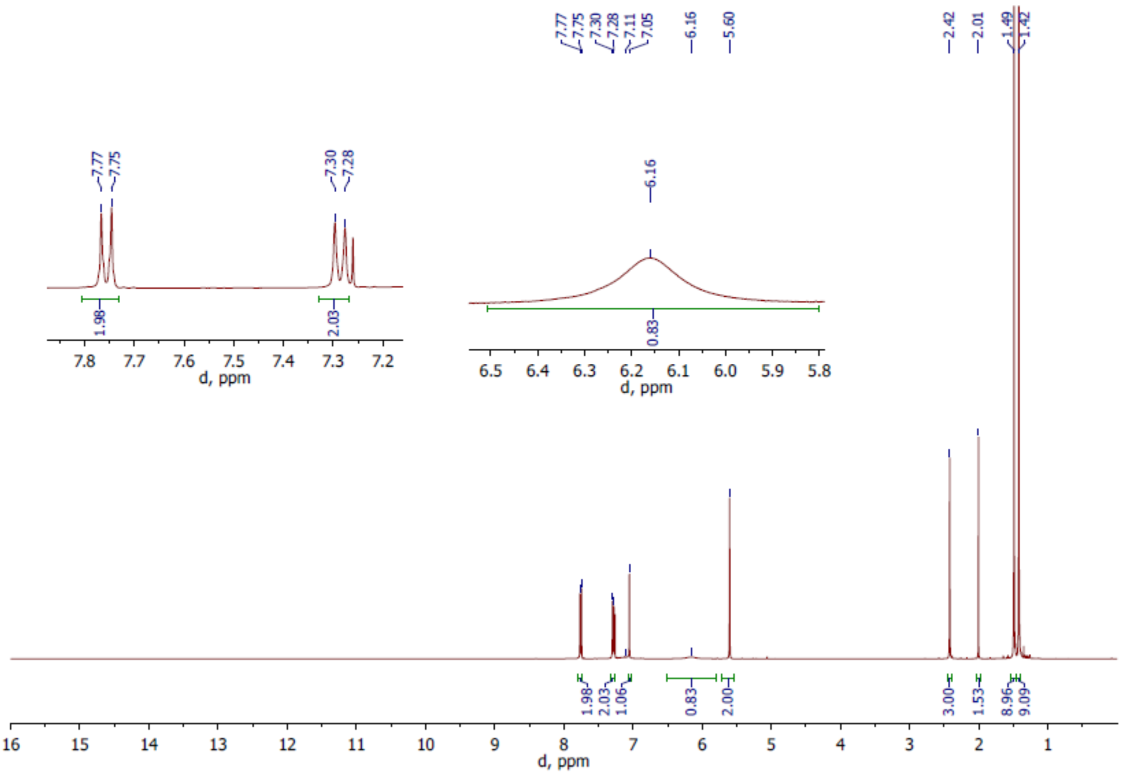

Figure S11. The ^{1}H NMR spectrum of 3-(4,6-di-*tert*-butyl-2,3-dihydroxybenzyl)-5-*p*-tolyl-1,3,4-oxadiazole-2(3H)-thione (**6**) (400 MHz, $CDCl_3$).

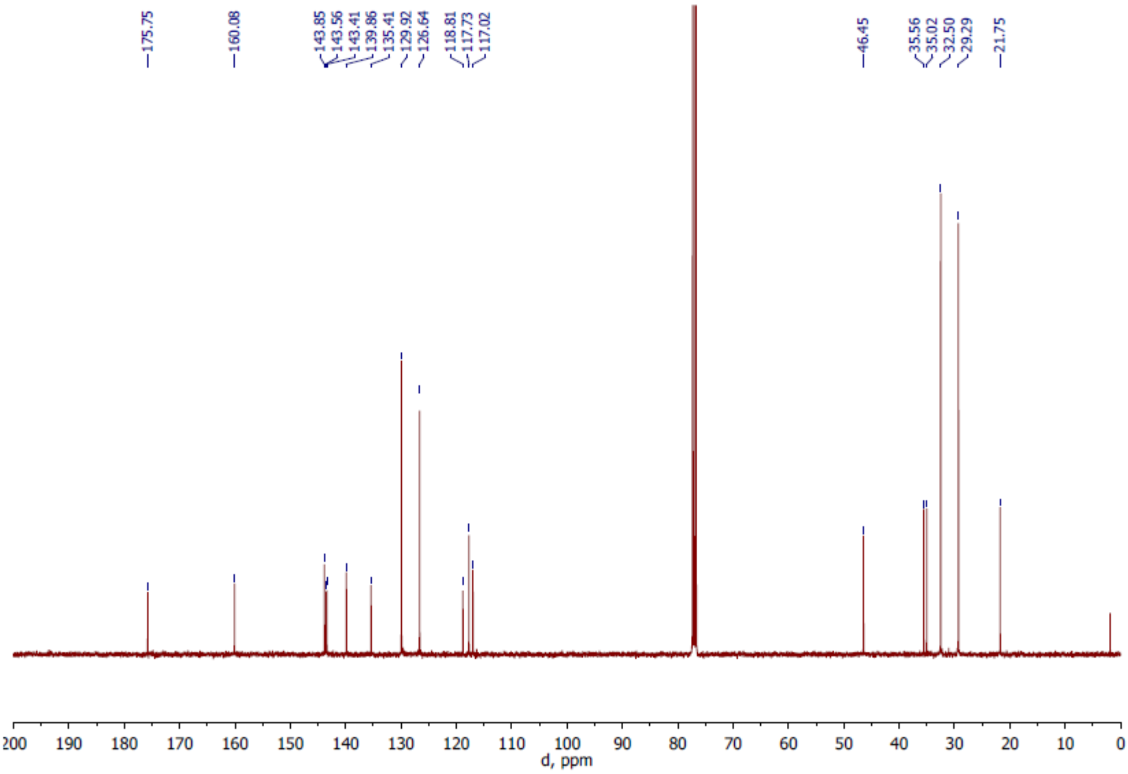

Figure S12. The $^{13}C\{^{1}H\}$ NMR spectrum of 3-(4,6-di-*tert*-butyl-2,3-dihydroxybenzyl)-5-*p*-tolyl-1,3,4-oxadiazole-2(3H)-thione (**6**) (100 MHz, $CDCl_3$).

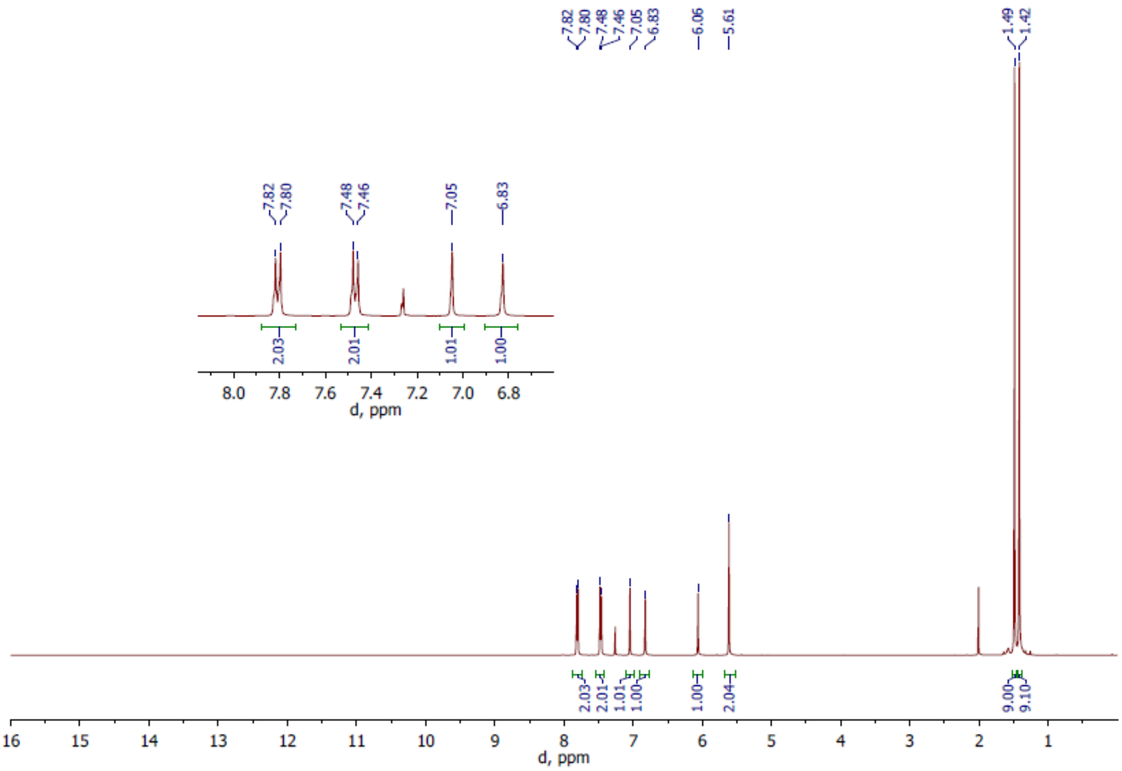

Figure S13. The ^{1}H NMR spectrum 5-(4-chlorophenyl)-3-(4,6-di-*tert*-butyl-2,3-dihydroxybenzyl)-1,3,4-oxadiazole-2(3*H*)-thione (**7**) (400 MHz, $CDCl_3$).

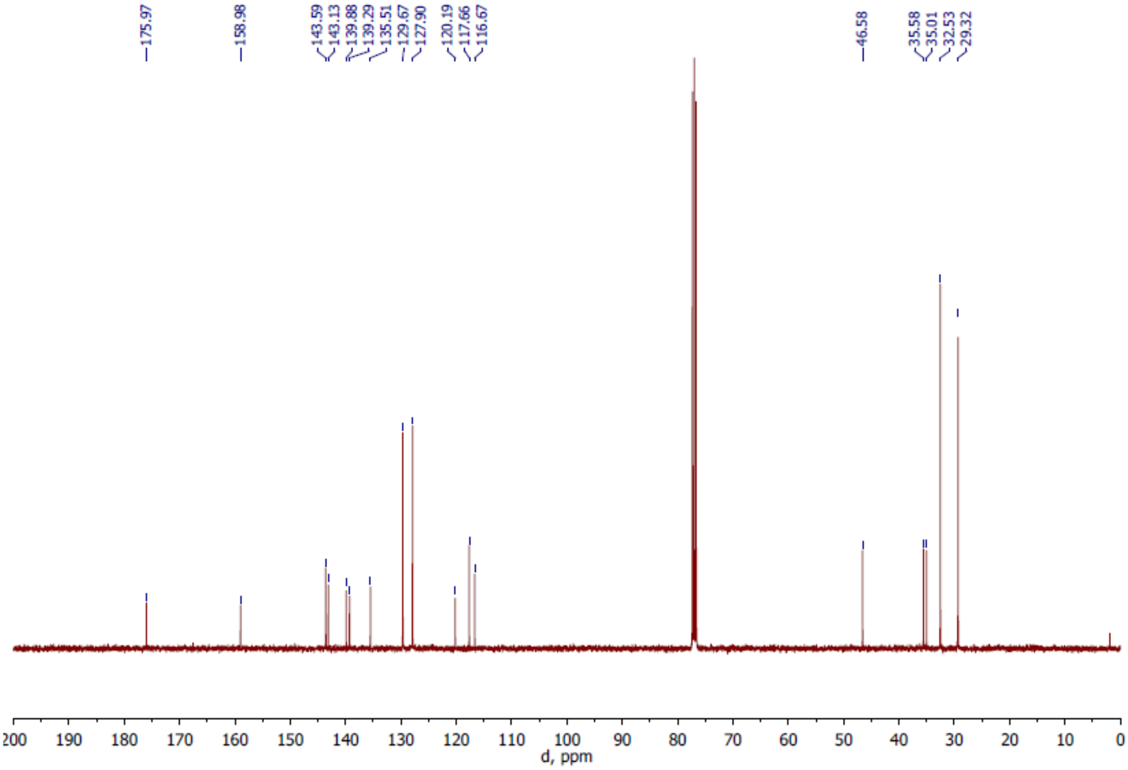

Figure S14. The ^{13}C{^{1}H} NMR spectrum of 5-(4-chlorophenyl)-3-(4,6-di-*tert*-butyl-2,3-dihydroxybenzyl)-1,3,4-oxadiazole-2(3*H*)-thione (**7**) (100 MHz, $CDCl_3$).

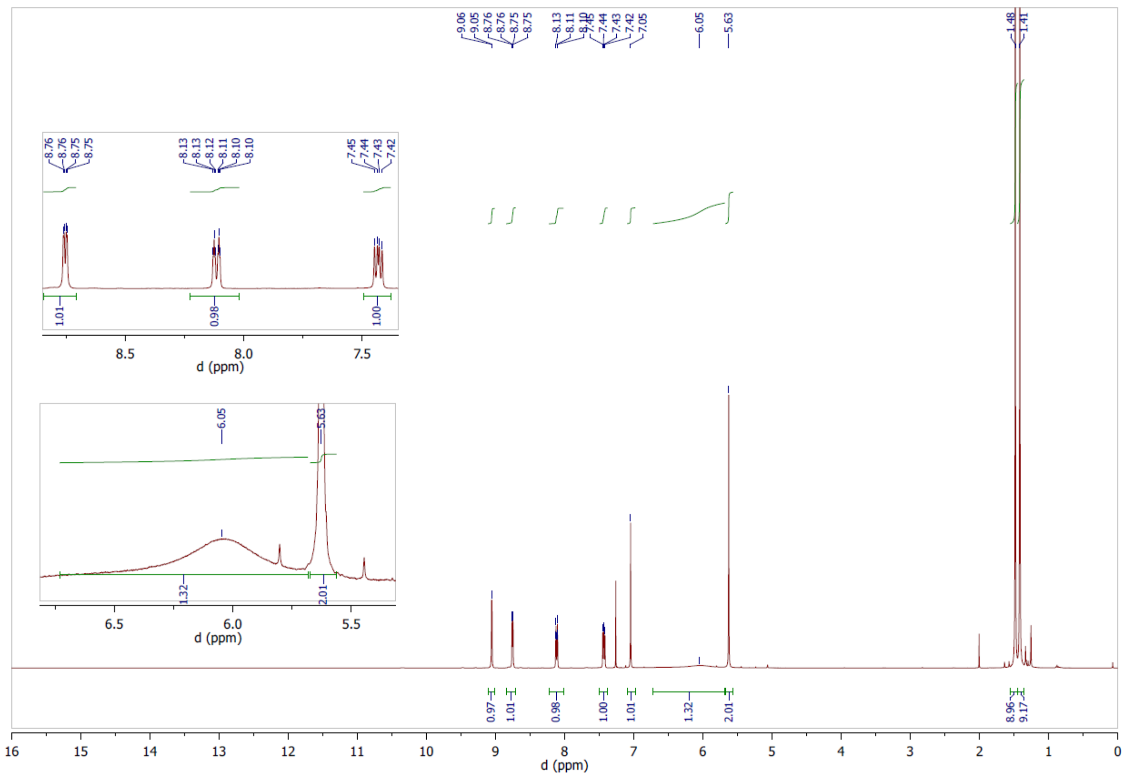

Figure S15. The ^{1}H NMR spectrum of 3-(4,6-di-*tert*-butyl-2,3-dihydroxybenzyl)-5-(pyridin-3-yl)-1,3,4-oxadiazole-2(3*H*)-thione (**8**) (400 MHz, $CDCl_3$).

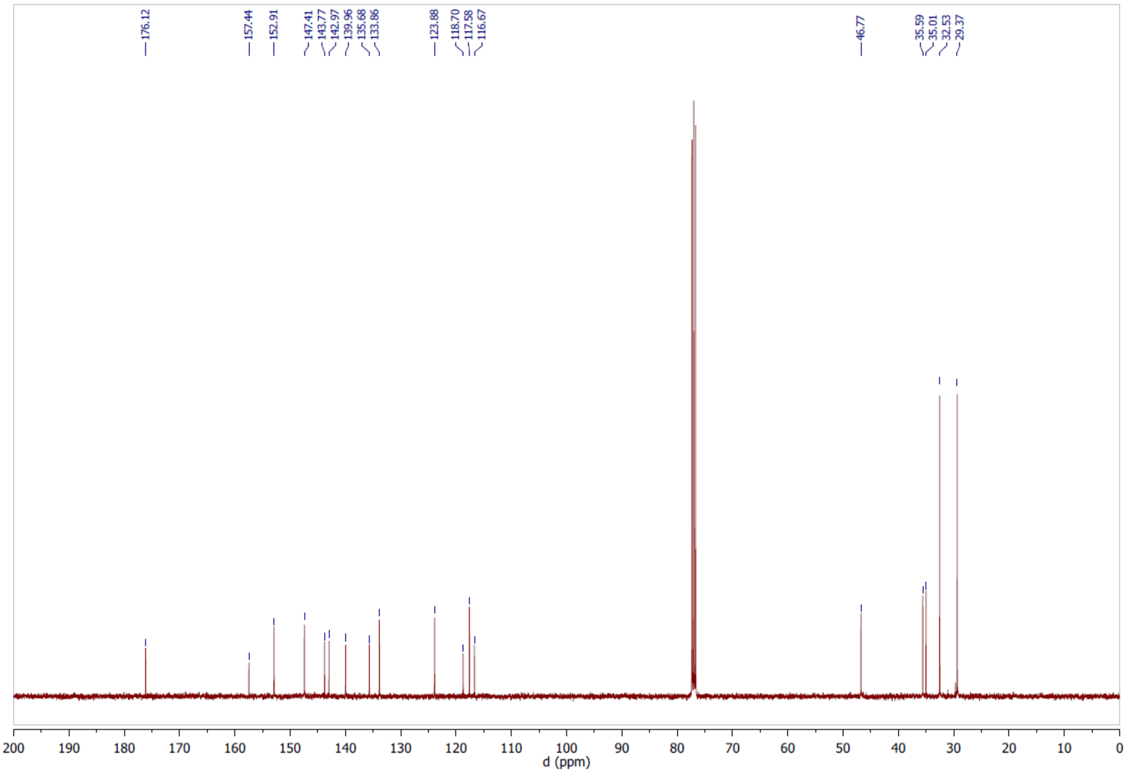

Figure S16. The $^{13}C\{^1H\}$ NMR spectrum of 3-(4,6-di-*tert*-butyl-2,3-dihydroxybenzyl)-5-(pyridin-3-yl)-1,3,4-oxadiazole-2(3*H*)-thione (**8**) (100 MHz, $CDCl_3$).

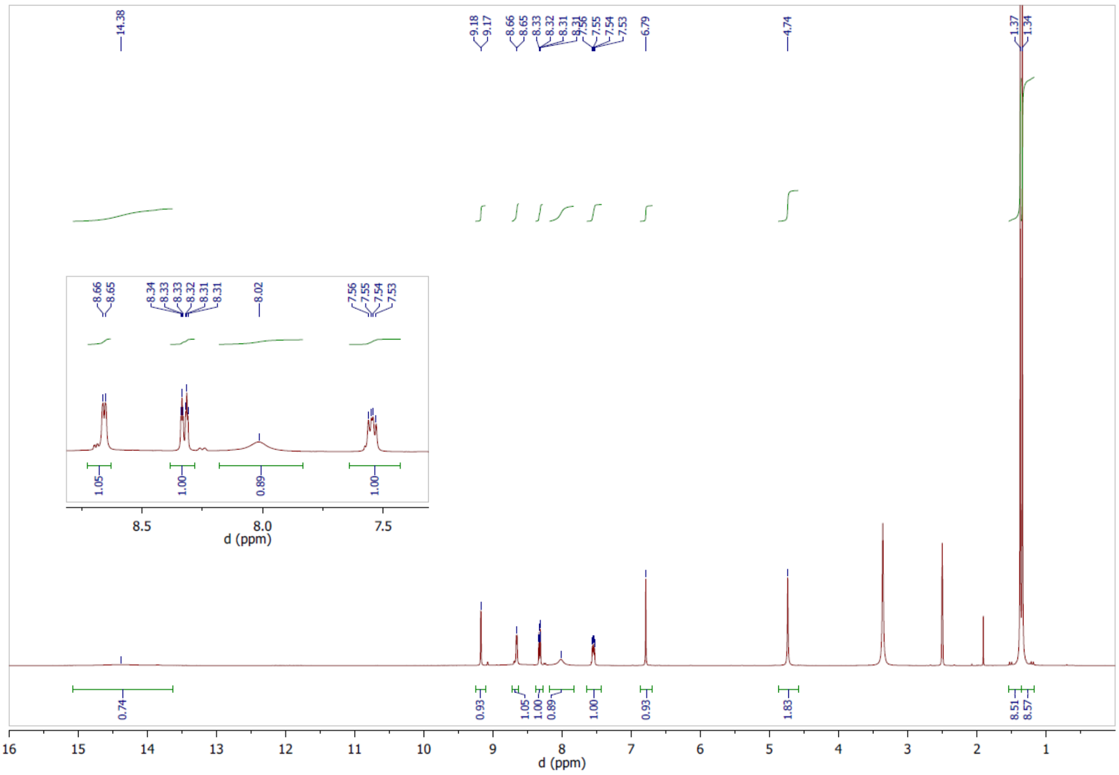

Figure S17. The ^{1}H NMR spectrum of 1-(4,6-di-*tert*-butyl-2,3-dihydroxybenzyl)-3-(pyridin-3-yl)-1*H*-1,2,4-triazole-5(4*H*)-thione (**9**) (400 MHz, DMSO-d_6).

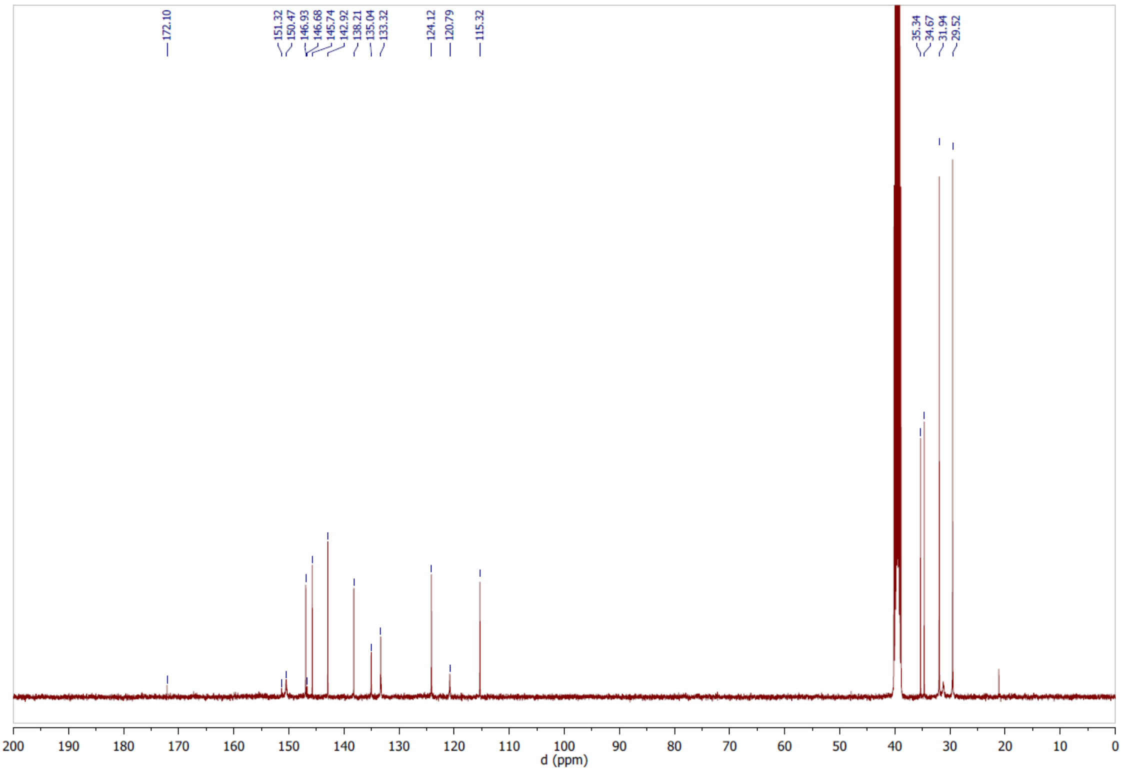

Figure S18. The $^{13}C\{^1H\}$ NMR spectrum of 1-(4,6-di-*tert*-butyl-2,3-dihydroxybenzyl)-3-(pyridin-3-yl)-1*H*-1,2,4-triazole-5(4*H*)-thione (**9**) (100 MHz, DMSO-d_6).

S3. Electrochemical data

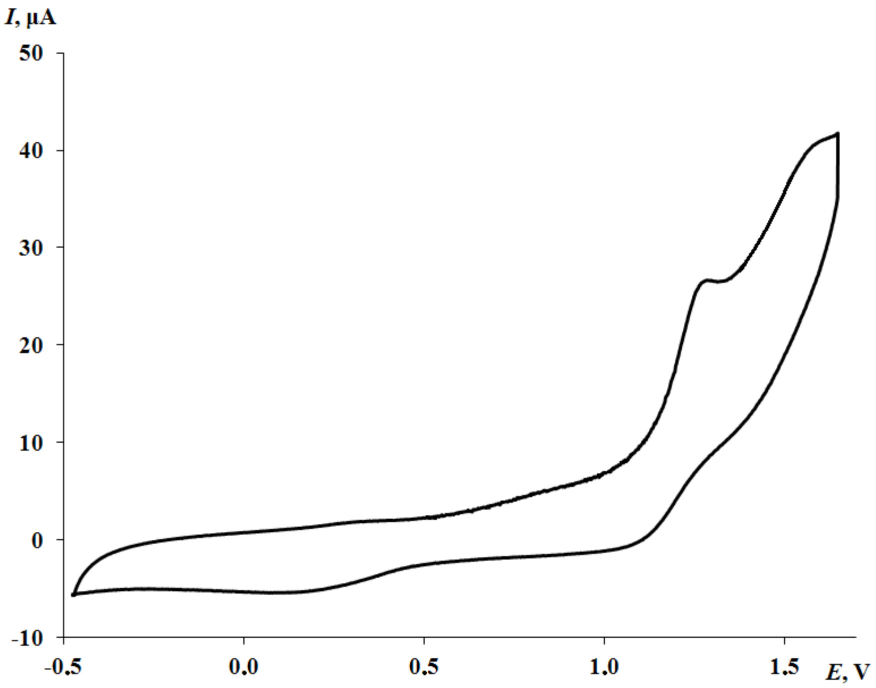

Figure S19. The CV curve of **1** at the potential ranges from −0.50 to 1.65 V (CH_3CN, GC electrode, Ag/AgCl/KCl(sat.), 0.15 M *n*-Bu_4NClO_4, *c* = 1 mmol·L^{-1}).

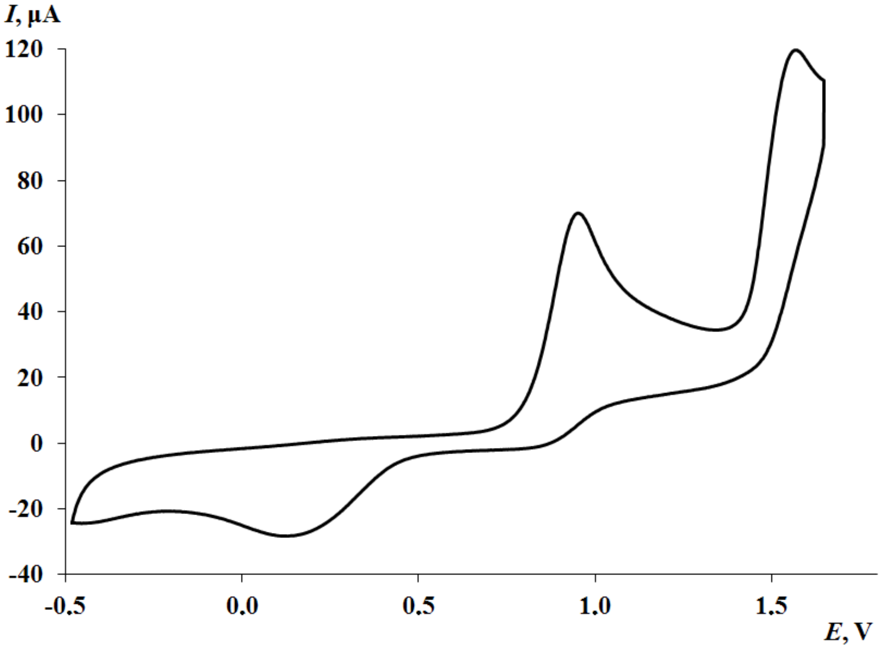

Figure S20. The CV curve of **4** at the potential ranges from −0.50 to 1.65 V (CH_3CN, GC electrode, Ag/AgCl/KCl(sat.), 0.15 M *n*-Bu_4NClO_4, *c* = 3 mmol·L^{-1}).

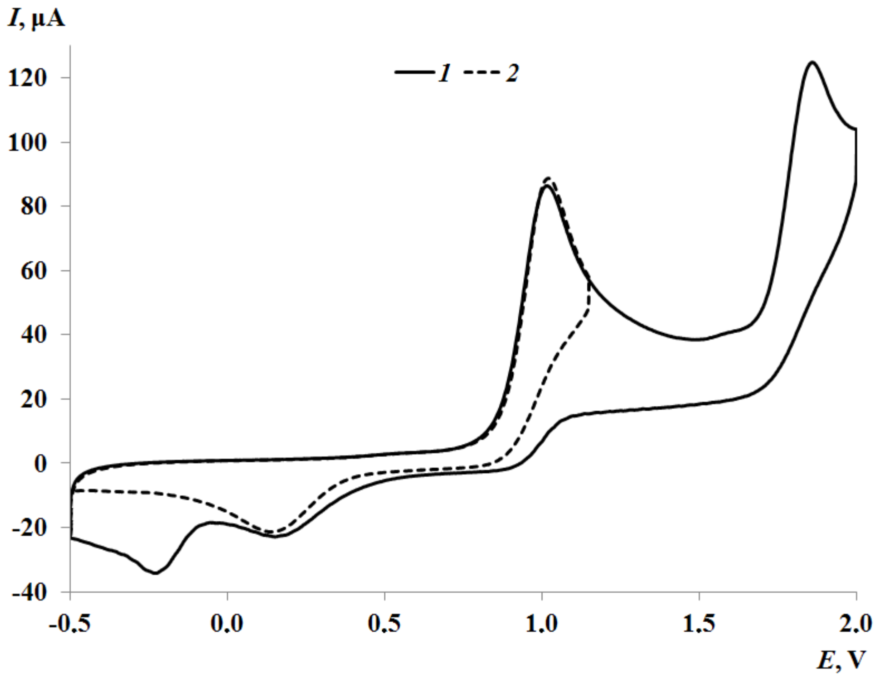

Figure S21. The CV curves of **5** at the potential ranges from –0.5 to 2.0 V (curve 1); from −0.5 to 1.2 V (curve 2) (CH_3CN, GC electrode, Ag/AgCl/KCl(sat.), 0.15 M *n*-Bu_4NClO_4, c = 3 mmol·L^{-1}).

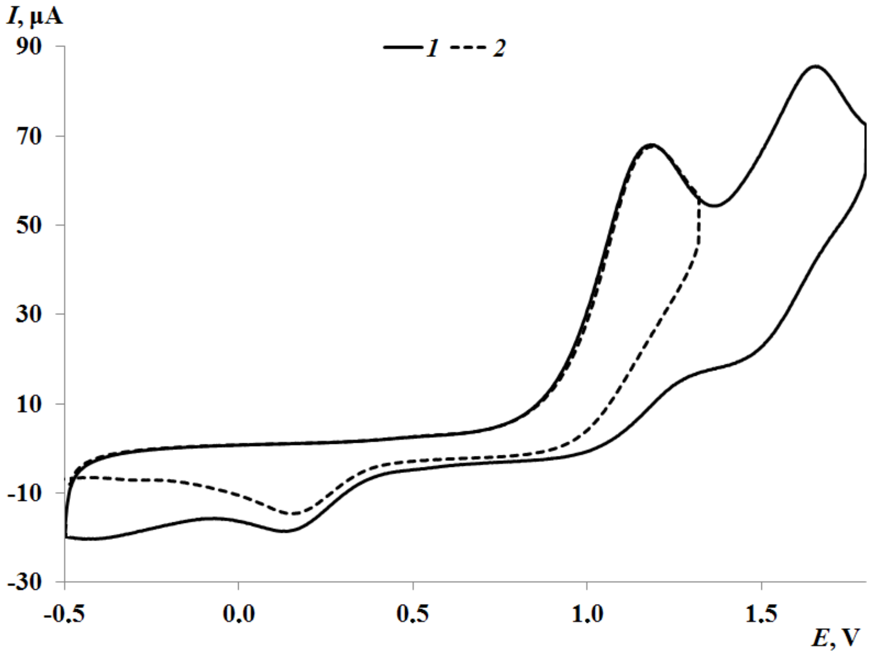

Figure S22. The CV curves of **6** at the potential ranges from −0.5 to 1.7 V (curve 1); from −0.5 to 1.3 V (curve 2) (CH_3CN, GC electrode, Ag/AgCl/KCl(sat.), 0.15 M *n*-Bu_4NClO_4, c = 3 mmol·L^{-1}).

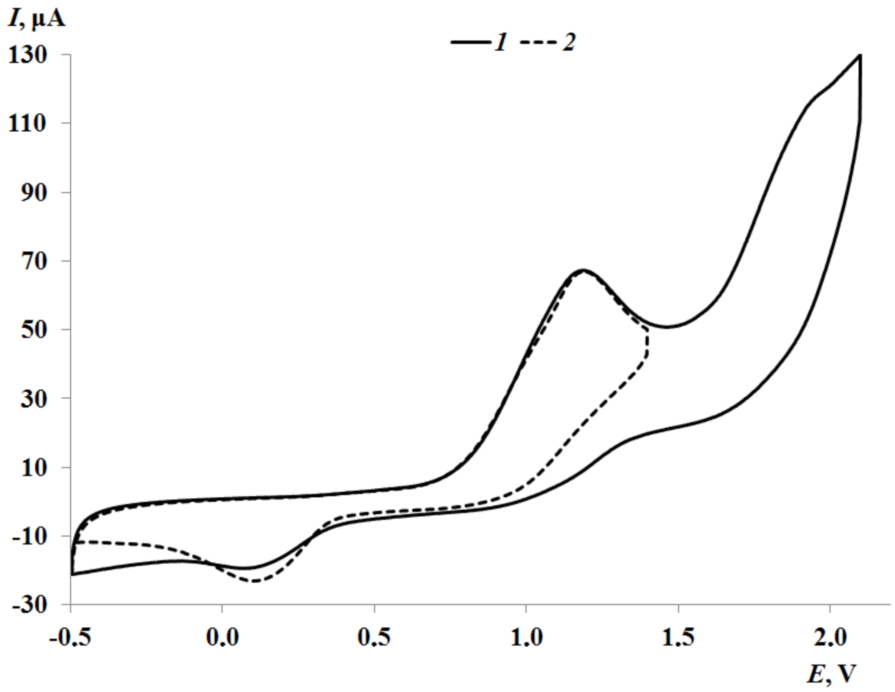

Figure S23. The CV curves of **8** at the potential ranges from −0.5 to 2.1 V (curve 1); from −0.5 to 1.4 V (curve 2) (CH_3CN, GC electrode, Ag/AgCl/KCl(sat.), 0.15 M *n*-Bu_4NClO_4, *c* = 3 mmol·L^{-1}).

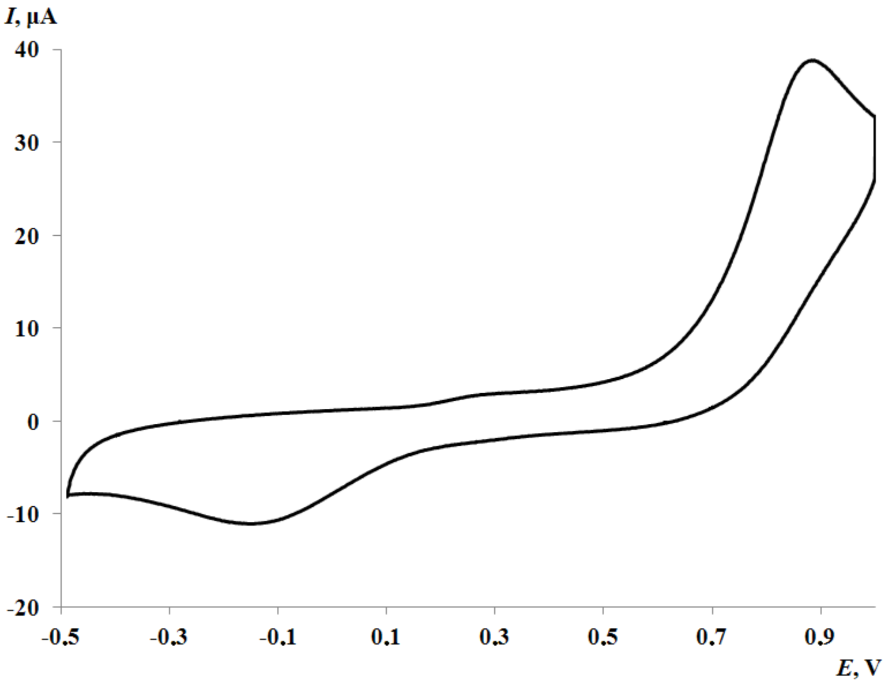

Figure S24. The CV curve of **9** at the potential ranges from −0.5 to 1.0 V (CH_3CN–DMSO, GC electrode, Ag/AgCl/KCl(sat.), 0.15 M *n*-Bu_4NClO_4, *c* = 3 mmol·L^{-1}).

S4. References

1. Arsenyev, M.V., Baranov, E.V., Shurygina, M.P., Chesnokov, S.A., Abakumov, G.A. *Mend. Commun.* **2016**, *26 (6)*, 552–554. https://doi.org/10.1016/j.mencom.2016.11.032.
2. Gordon, A.J., Ford, R.A. The chemist’s companion. A Wiley interscience publication, New York, **1972**, 541 pp.
3. Smolyaninov, I.V., Pitikova, O.V., Poddel’sky, A.I., Berberova, N.T. *Russ. Chem. Bull.* **2018**, *67*, 1857–1867. https://doi.org/10.1007/s11172-018-2299-9.
4. Krause, L., Herbst-Irmer, R., Sheldrick, G.M., Stalke, D. *J. Appl. Crystallogr.* **2015**, *48 (1)*, 3–10. https://doi.org/10.1107/S1600576714022985.
5. CrysAlisPro, version 1.171.38.41; Rigaku Oxford Diffraction. **2015**.
6. Dolomanov, O.V., Bourhis, L.J., Gildea, R.J., Howard, J.A.K., Puschmann, H. J. *Appl. Crystallogr.* **2009**, *42 (2)*, 339–341. https://doi.org/10.1107/S0021889808042726.
7. Sheldrick, G.M. *Acta Cryst. A Found. Crystallograp.* **2008**, *64 (1)*, 112–122. https://doi.org/10.1107/S0108767307043930.
8. Sheldrick, G.M. *Acta Crysta. C Struct. Chem.* **2015**, *71 (1)*, 3–8. https://doi.org/10.1107/S2053229614024218.
9. Sheldrick, G.M., *Acta Cryst. A Found. Adv.* **2015**, *71 (1)*, 3–8. https://doi.org/10.1107/S2053273314026370.
10. Bondet, V., Brand-Williams, W., Berset, C. *LWT - Food Sci.Technol.* **1997**, *30 (6)*, 609–615. https://doi.org/10.1006/fstl.1997.0240.
11. Smolyaninov, I.V., Antonova, N.A., Poddel’sky, A.I., Smolyaninova, S.A., Osipova, V.P., Berberova, N.T. *J. Organomet. Chem.* **2011**, *696 (13)*, 2611–2620. https://doi.org/10.1016/j.jorganchem.2011.04.004.

12. Re, R., Pellergrini, N., Proteggente, A., Pannala, A., Yang, M., Rice-Evans, C. *Free Radic. Biol. Med.* **1999**, *26*, 1231–1237. https://doi.org/10.1016/S0891-5849(98)00315-3.

13. Smolyaninov, I.V., Poddel'sky, A.I., Burmistrova, D.A., Voronina, Y.K., Pomortseva, N.P., Polovinkina, M.A., Almyasheva, N.R., Zamkova, M.A., Berberova, N.T., Eremenko, I.L. *Int. J. Mol. Sci.* **2023**, *24 (9)*, 8319. https://doi.org/10.3390/ijms24098319.

14. Özyürek, M., Güçlü, K., Tütem, E., Başkan, K.S., Erçağ, E., Esin Çelik, S., Baki, S., Yıldız, L., Karaman, Ş., & Apak, R. *Anal. Methods* **2011**, *3 (11)*, 2439. https://doi.org/10.1039/c1ay05320e.

15. Smolyaninov, I.V., Burmistrova, D.A., Arsenyev, M.V., Polovinkina, M.A., Pomortseva, N.P., Fukin, G.K., Poddel'sky, A.I., Berberova, N.T. *Molecules* **2022**, *27 (10)*, 3169. https://doi.org/10.3390/molecules27103169.

16. Stroev, E.N., Makarova, V.G., Matveeva, I.V. Praktikum po biologiheskoy khimii [Practical Work in Biological Chemistry], MIA, Moscow, **2012**, 384 pp. (in Russian).
